# Synergistic interaction between microbial nitrogen fixation and iron reduction in the environment

**DOI:** 10.1093/ismejo/wraf212

**Published:** 2025-09-23

**Authors:** Xiaohan Liu, Ping Li, Keman Bao, Yaqi Wang, Helin Wang, Yanhong Wang, Zhou Jiang, Yi Yang, Songhu Yuan, Andreas Kappler, Yanxin Wang

**Affiliations:** State Key Laboratory of Geomicrobiology and Environmental Changes, China University of Geosciences, Wuhan, Hubei 430074, P.R. China; Hubei Key Laboratory of Yangtze Catchment Environmental Aquatic Science, School of Environmental Studies, China University of Geosciences, Wuhan, Hubei 430074, P.R. China; State Key Laboratory of Geomicrobiology and Environmental Changes, China University of Geosciences, Wuhan, Hubei 430074, P.R. China; Hubei Key Laboratory of Yangtze Catchment Environmental Aquatic Science, School of Environmental Studies, China University of Geosciences, Wuhan, Hubei 430074, P.R. China; State Key Laboratory of Geomicrobiology and Environmental Changes, China University of Geosciences, Wuhan, Hubei 430074, P.R. China; Hubei Key Laboratory of Yangtze Catchment Environmental Aquatic Science, School of Environmental Studies, China University of Geosciences, Wuhan, Hubei 430074, P.R. China; State Key Laboratory of Geomicrobiology and Environmental Changes, China University of Geosciences, Wuhan, Hubei 430074, P.R. China; Hubei Key Laboratory of Yangtze Catchment Environmental Aquatic Science, School of Environmental Studies, China University of Geosciences, Wuhan, Hubei 430074, P.R. China; State Key Laboratory of Geomicrobiology and Environmental Changes, China University of Geosciences, Wuhan, Hubei 430074, P.R. China; Hubei Key Laboratory of Yangtze Catchment Environmental Aquatic Science, School of Environmental Studies, China University of Geosciences, Wuhan, Hubei 430074, P.R. China; State Key Laboratory of Geomicrobiology and Environmental Changes, China University of Geosciences, Wuhan, Hubei 430074, P.R. China; Hubei Key Laboratory of Yangtze Catchment Environmental Aquatic Science, School of Environmental Studies, China University of Geosciences, Wuhan, Hubei 430074, P.R. China; State Key Laboratory of Geomicrobiology and Environmental Changes, China University of Geosciences, Wuhan, Hubei 430074, P.R. China; College of Marine Science and Technology, China University of Geosciences, Wuhan, Hubei 430074, P.R. China; State Key Laboratory of Geomicrobiology and Environmental Changes, China University of Geosciences, Wuhan, Hubei 430074, P.R. China; Hubei Key Laboratory of Yangtze Catchment Environmental Aquatic Science, School of Environmental Studies, China University of Geosciences, Wuhan, Hubei 430074, P.R. China; Department of Geosciences, University of Tübingen, Tübingen, Baden-Württemberg 72074, Germany; Cluster of Excellence: EXC 2124: Controlling Microbes to Fight Infection, University of Tübingen, Tübingen, Baden-Württemberg 72074, Germany; State Key Laboratory of Geomicrobiology and Environmental Changes, China University of Geosciences, Wuhan, Hubei 430074, P.R. China; Hubei Key Laboratory of Yangtze Catchment Environmental Aquatic Science, School of Environmental Studies, China University of Geosciences, Wuhan, Hubei 430074, P.R. China

**Keywords:** nitrogen fixation, iron reduction, ^15^N isotope tracing, transcriptomics, metagenomics

## Abstract

Nitrogen and iron are essential yet often limiting nutrients in many ecosystems. Microbial nitrogen fixation by diazotrophs and dissimilatory ferric iron reduction are key processes that sustain nitrogen and iron availability. However, their interactions are not well understood. Here, we demonstrate a synergistic relationship between microbial nitrogen fixation and ferric iron reduction, observed in both laboratory cultures and environmental samples. In diazotrophic ferric iron-reducing bacteria, including *Klebsiella grimontii* N7 and *Geobacter sulfurreducens* PCA, nitrogen fixation enhanced heterotrophic ferric iron-reducing rates by 14.7- and 2.69-fold, respectively, and ferric iron reduction concurrently increased ^15^N_2_ fixation by up to 100%. A similar synergy was observed in an interspecies system comprising the diazotroph *Azospirillum humicireducens* SgZ-5T and the dissimilatory ferric iron-reducing bacterium *Shewanella oneidensis* MR-1. Transcriptomic analysis revealed that nitrogen fixation upregulated pathways involved in carbon and nitrogen metabolism, including amino acid biosynthesis, glycolysis, and the tricarboxylic acid cycle (*P* < 0.01), thereby accelerating ferric iron reduction through nitrogen supply. In turn, ferric iron reduction stimulated organic carbon oxidation, generating the energy, and reducing equivalents needed for microbial nitrogen fixation. These findings were further validated through microcosm experiments and meta-omics analyses of environmental samples from aquifers, marine sediments, hot springs, and soils, providing new insights into the coupled nitrogen, iron, and carbon cycles in natural ecosystems.

## Introduction

Nitrogen (N) [1] and iron (Fe) [2] are fundamental elements that govern microbial activity and biogeochemical cycling in natural ecosystems. Although dinitrogen gas (N_2_) constitutes the major reservoir of N on Earth, it must first be biologically converted into ammonium (NH_4_^+^) by diazotrophs to become bioavailable [3]. Microbial nitrogen fixation (MNF) thus plays a critical role in sustaining ecosystem productivity, contributing nearly 50% of the global N input [4]. Unlike the Haber–Bosch process (the industrial fixation of N_2_ into NH_4_^+^) [5, 6], which is energy-intensive and a major contributor to greenhouse gas emissions [7–9], MNF occurs naturally [10] in diverse habitats such as marine sediments [11], estuaries [12], soils [13], and aquifers [14].

Because MNF demands large amounts of high-potential electrons and adenosine triphosphate (ATP) [15], diazotrophs often couple MNF with other metabolic pathways, particularly those involved in sulfur (S) and carbon (C) cycling [16]. For example, MNF has been shown to co-occur with sulfate reduction, sulfur oxidation, and methane (CH_4_) metabolism in rhizospheres [17], coastal systems [18], and methane seeps [19], reflecting the metabolic flexibility and ecological diversity of diazotrophs [20]. Moreover, strong positive correlations between MNF activity and dissolved organic carbon (DOC) concentrations in aquatic environments [21] suggest that organic matter (OM) availability is a key factor in supporting MNF [22].

Dissimilatory ferric (Fe(III)) reduction (DIR) is a widespread anaerobic respiratory process that drives OM oxidation and C turnover in sediments and subsurface environments [2, 23]. Given that DIR facilitates OM degradation, it might indirectly contribute to MNF by enhancing the availability of energy and electron donors. Conversely, MNF produces bioavailable N, which could enhance microbial growth and activity [24], thereby reinforcing DIR. Thus, we hypothesize that MNF and DIR are metabolically interconnected and mutually reinforcing. Although previous studies have reported that the addition of Fe(III) (oxyhydr)oxides can stimulate nitrogenase activity in paddy soils [25], and that several DIR bacteria (DIRBs), including *Geobacter*, *Geomonas*, *Anaeromyxobacter*, and *Klebsiella*, also possess N_2_ fixation abilities [26–28], direct evidence for such interaction is still lacking, and its underlying mechanisms remain poorly understood.

To test the hypothesis that MNF and DIR synergistically enhance each other, we selected two types of diazotrophic DIRBs, *K. grimontii* N7 (fermentative) and *G. sulfurreducens* PCA (respiratory), as well as an interspecies system composed of a N-fixing bacterium (*A. humicireducens* SgZ-5T) and a DIRB (*S. oneidensis* MR-1). The environmental relevance of this synergy was further validated through microcosm incubations using sediments and soils collected from aquifers, marine environments, hot springs, and terrestrial ecosystems. A combination of quantitative RT-PCR (RT-qPCR), transcriptomic, metagenomic, and metatranscriptomic analyses of pure cultures and environmental samples was employed to investigate the evidence and elucidate the mechanisms of MNF-DIR synergy.

## Materials and methods

### Strains, environmental samples, and media

The strain *K. grimontii* N7 (N7), a facultatively anaerobic N-fixing bacterium, was isolated from groundwater as described in a previous study [14] (Fig. S1) (accession number PRJNA1160161). Strain N7 can reduce Fe(III)-citrate in N-free Burk’s medium. The diazotrophic DIRB *G. sulfurreducens* PCA was purchased from the American Type Culture Collection (Manassas, VA, USA) (ATCC 51573). *A. humicireducens* SgZ-5T (CCTCC AB 2012021), which can fix N in Burk’s medium, was purchased from the China Center for Type Culture Collection (Wuhan University, China). The DIRB *S. oneidensis* MR-1, which cannot fix N but can reduce Fe(III), was also obtained from ATCC (number: 700550). To activate cultures, Luria Broth (LB) medium containing (per liter) 10 g NaCl, 5 g yeast extract, and 10 g tryptone was used to grow *K. grimontii* N7 and *S. oneidensis* MR-1 at 30°C. *A. humicireducens* SgZ-5T was cultured in Nutrient Broth (NB) medium containing (per liter) 5 g NaCl, 5 g peptone, and 3 g beef extract at 30°C. Modified anoxic Burk’s medium was used to cultivate strains for both N fixation and DIR at 30°C. The medium contained the following components (per liter): 20 g mannitol, 0.2 g KH_2_PO_4_, 0.8 g·K_2_HPO_4_, 0.2 g MgSO_4_·7H_2_O, 0.1 g CaSO_4_·2H_2_O, and trace amounts of Na_2_MoO_4_·2H_2_O and FeCl_3_, at pH 7.0. Fe(III) reduction was conducted in this medium supplemented with Fe(III)-citrate. *G. sulfurreducens* PCA was cultured in an N-free FWAFC medium for N fixation and Fe(III) reduction analyses, supplemented with 20 mM sodium acetate as the electron donor and C source and 60 mM Fe(III)-citrate as the electron acceptor. The composition and preparation process of the medium is described in the previous study [28] and the Supplementary Materials Section S1.

Representative aquifer sediments were collected from two boreholes, XHC, LDS-20, and LDS-30, in the recharge (XHC) and discharge (LDS) areas of Hetao Plain, Inner Mongolia, China. Sampling depths were 20 m for XHC and LDS-20, and 30 m for LDS-30 [29]. Hot spring sediments were sampled from two drainage channels in Qamdo, eastern Tibet, designated as DB-A and DB-B [30]. Surface soil samples (0–10 cm) were collected from Jianghan Plain (JH1) [31] and Shenhu International Important Wetland (TSH and S07) in southeast Hubei Province, respectively. Additionally, three marine sediments (A7-1, A7-7, and C4) were obtained from the surface (0–5 cm layer) of a box core in the East China Sea. Basic information is summarized for these samples (Table S1). All fresh sediments and soils were sealed in 50 mL sterile polyethylene tubes, stored on dry ice, and transported to the laboratory for homogenization and microcosmic culturing within 3 days after collection.

### Monoculture and interspecies assays

Strain *K. grimontii* N7 was routinely cultured in 150 mL of LB medium at 30°C with shaking at 150 rpm. Cells were harvested by centrifugation (8,000 rpm, 10 min, 4°C), washed three times with phosphate-buffered saline (PBS), and resuspended in sterilized anoxic N- and Fe-free Burk’s medium. The cell suspension was adjusted to an OD_600_ of 0.05 and inoculated into fresh Burk’s medium for downstream assays. To investigate the potential synergy between MNF and DIR, four experimental groups were established: Burk, Burk+Fe, N_2_ + Fe, and Ar + Fe. The N_2_ + Fe and Ar + Fe groups were supplemented with 5 mM Fe(III)-citrate and degassed with N_2_ and argon gas (Ar), respectively, to assess the impact of MNF on DIR. For Burk+Fe cultures simulating environmentally relevant Fe levels, 0.2 mM Fe(III)-citrate was used. A noninoculated group was included as a blank control. Additionally, the interaction between biological N_2_O fixation and DIR was evaluated using ^15^N-N_2_O tracing experiments. Details of the experimental design are provided in Supplementary Materials Section S2. *G. sulfurreducens* PCA was cultured anaerobically in N-free FWAFC medium. At the early stationary phase (OD_600_ ≈ 0.5), 10 mL of culture was transferred into 100 mL of N-free FWAFC medium. The N_2_ + Fe and Ar + Fe groups were degassed with 80% N_2_–20% CO_2_ and 80% Ar-20% CO_2_, respectively. Treatments with and without Fe(III)-citrate were used to evaluate the effect of DIR on MNF. All cultures were incubated at 30°C in the dark in triplicate.

A co-culture system consisting of diazotroph *A. humicireducens* SgZ-5T and DIRB *S. oneidensis* MR-1 was constructed to explore interspecies interactions between MNF and DIR. Active cells from both strains were harvested, washed three times with PBS, and resuspended in sterilized anoxic N/Fe-free Burk’s medium. The suspensions of diazotroph and DIRB were inoculated either individually or in combination (at a 1:1 volume ratio, each with an OD_600_ of 0.02). The noninoculated treatment served as the blank control. All groups were supplemented with 5 mM Fe(III)-citrate, degassed with N_2_, and incubated at 30°C with shaking at 150 rpm in the dark.

### Characterization of nitrogen fixation and Fe(III) reduction

Nitrogenase activity was characterized using acetylene-reduction assays as previously described [32]. Briefly, 3 mL headspace air (10% of headspace) was extracted from the anoxic medium and replaced with 3 mL acetylene. The ethylene concentration was measured by a gas chromatograph (GC-4000A, EWAI) equipped with a flame ionization detector and a Porpack N column. The concentrations of fixed N in various treatment group solutions were determined using a ^15^N isotope tracing technique. Briefly, 60 mL serum bottles containing 30 mL of medium were degassed with Ar for 40 min, after which the headspace was replaced with 30 mL ^15^N_2_ (>98 atom%, Cambridge Isotope Laboratories). The bottles were incubated inverted at 30°C with shaking at 150 rpm. After incubation, the cultures were immediately preserved with 1 mL of saturated HgCl_2_ solution and purged with Ar for 40 min to remove residual ^15^N_2_. The fixed ^15^N-labeled products (including NH_4_^+^ and organic N fractions) were oxidized with hypobromite iodine solution and analyzed as ^29^N_2_ and/or ^30^N_2_ using a membrane inlet mass spectrometer (MIMS, Hiden HPR-40, UK) [12, 33, 34]. A standard calibration curve was generated using a concentration gradient of ^15^NH_4_Cl (99%, Cambridge Isotope Laboratories). The level of ^15^N labeling was linearly correlated with the total production of ^29^N_2_ and ^30^N_2_ signals (Fig. S2). Initial sample bottles were immediately inactivated after ^15^N_2_ tracer addition, and the resulting ^15^N signal was measured as the *t_0_* control.

For Fe(II) and total Fe quantification, samples were collected under anoxic conditions in a glovebox, mixed with an equal volume of 1 M HCl, and analyzed using a ferrozine assay [35]. DOC was measured by filtering the bacterial suspension through 0.22-μm membrane filters (Millipore) and analyzing with a total organic carbon (TOC) analyzer (TOC-L, Shimadzu, Japan). The N_2_O and CO_2_ content in the headspace was determined using a gas chromatograph (Shimadzu GC-2014). Amino Acid (AA) production during N fixation was quantified using an AA Assay Kit (Sangon, China). Intracellular ATP levels were measured according to the manufacturer’s instructions using an ATP Content Assay Kit (Solarbio Science & Technology Co., Ltd) [36]. Mannitol content was determined using spectrophotometry [37].

### RNA extraction, RT-qPCR, and transcriptomic analysis

To investigate the gene expression of strains during MNF and DIR, three treatment groups of intraspecific assays were analyzed via transcriptomics (N7): Burk, Burk+Fe, and Burk+Fe + Ar. Both Burk and Burk+Fe groups were purged with N_2_ gas, whereas 0.2 mM Fe(III)-citrate was added to the Burk+Fe group. The Burk+Fe + Ar group, also treated with 0.2 mM Fe(III)-citrate, was degassed with Ar. Details on RNA sampling for transcriptomics are provided in Section S3 of the Supplementary Materials. In cross-feeding experiments, gene expression was profiled for three groups: monocultures of *A. humicireducens* SgZ-5T and *S. oneidensis* MR-1, and their co-culture. Following incubation, cells in all treatments were collected during the exponential phase via centrifugation (8,000 rpm, 20 min, 4°C). Total RNA was extracted using RNAiso Plus (TaKaRa, Bio) following the manufacturer’s protocol, and residual DNA in RNA was removed with the PrimeScript RT reagent Kit with gDNA Eraser (TaKaRa, Bio) for 2 min at 42°C. RNA integrity was assessed via 1.5% agarose gel electrophoresis (Biowest, Riverside, MO, USA), and RNA concentrations were determined using a NanoDrop-2000 spectrophotometer (Thermo Fisher Scientific, Waltham, MA, USA). For cDNA synthesis, the RNA was diluted and reverse-transcribed using the PrimeScript RT reagent Kit with gDNA Eraser (TaKaRa, Bio). Quantitative RT-PCR (RT-qPCR) was then performed (Applied Biosystems, Waltham, MA, USA) with the TB Green Premix Ex TaqTM II (TaKaRa, Bio) qPCR kit. Details of the primers and thermal cycling conditions employed in amplification protocols are provided (Table S2). Transcript levels of *nifH*, *cymA*, *mtrA*, and the *Shewanella* 16S rRNA gene were quantified using the 2^−ΔΔCT^ method [38], with the bacterial 16S rRNA gene serving as the endogenous reference.

RNA samples were extracted and preserved at −80°C, then shipped on dry ice to Magigene Technology (Guangzhou, China) within 48 h for RNA-Seq library preparation using the ALFA-SEQ RNA Library Prep Kit II. Sequencing was performed on a NovaSeq 6000 System (Illumina). Raw reads were processed to remove low-quality data using Trimmomatic (Version 0.36) to generate clean reads [39] (Table S3). These were subsequently mapped to the reference genome of *K. grimontii* from the Kyoto Encyclopedia of Genes and Genomes (KEGG) database using Bowtie2 (Version 2.4.5) [40]. Quantification of read counts was performed using Salmon (Version 1.9.0), and differential expression analysis was conducted with DESeq 2 (Version 1.30.1) to identify differentially expressed genes (DEGs) [41, 42]. DEGs were considered significant with a *P*-adjust value <0.01 and |log_2_ fold change| ≥0.5. Gene annotation was performed using the KEGG database, and Gene Ontology (GO) enrichment analysis followed the protocol from our previous study [43].

### Sediment microcosms and metagenomic/metatranscriptomic analysis

To assess the environmental universality of the synergistic interaction between MNF and DIR, microcosm experiments were performed using three representative samples collected from aquifer sediments (XHC, LDS-20, and LDS-30, our previous study) [29], marine sediments (A7-1, A7-7, and C4), hot spring sediments (DB-A-1, DB-A-3, and DB-B-3, our unpublished data), and soils (JH1, S07, and TSH). Each microcosm was prepared in triplicate in 120 mL sterile serum bottles, containing 50 mL of slurry made by homogenizing sediment or soil with sterile water at a 1:5 (w/w) ratio. Two treatments were used to assess the influence of MNF on DIR: flushed with either N_2_ or Ar gas for 50 min. To evaluate whether DIR could stimulate MNF, two additional Fe(III)-amended treatments were included, using Fe(III)-citrate at concentrations representative of near-field Fe levels (Table S1). Hot spring sediments were incubated at 60°C, whereas all other microcosms were incubated at 25°C in the dark. Nitrogenase activity was measured using the acetylene-reduction assay (see Section 2.3). For ^15^N_2_ assimilation analysis, sediment and soil samples were lyophilized, ground, and packaged in tin cups for total N and ^15^N analysis using an element analyzer coupled with an isotope ratio mass spectrometer (EA Isolink-253 Plus, Thermo Fisher Scientific, Waltham, MA, USA). Fe(II) concentrations were measured under an anoxic glovebox. Supernatants from centrifuged samples (12,000 rpm, 5 min) were acidified in 1 M HCl and analyzed using the ferrozine assay. Sediment-associated Fe(II) was extracted with 0.5 M HCl and quantified similarly.

Metagenomic and metatranscriptomic analyses were carried out on the same representative microcosms. Due to the low biomass in hot spring sediments, only metagenomic data were obtained for this habitat. For a broader ecological context, we also incorporated public metagenomic datasets from aquifers (our previous study) [29], paddy soils [44–46], and marine waters/sediments [47, 48] (Fig. S3). Details on DNA/RNA extraction, sequencing data processing, and metagenomic binning procedures are provided in Supplementary Materials Section S4 and our recent publication [14]. Assembly statistics for metagenomic and metatranscriptomic data were evaluated for various environmental samples (Tables S4 and S5).

### Statistical analyses

Spearman correlations among the abundances of functional genes were computed using R v4.1.2. Data visualization, fitting, and evaluation were performed using GraphPad Prism v10 (GraphPad Software, San Diego, CA, USA).

## Results

### Effects of nitrogen fixation on Fe(III) reduction

**Figure 1:**
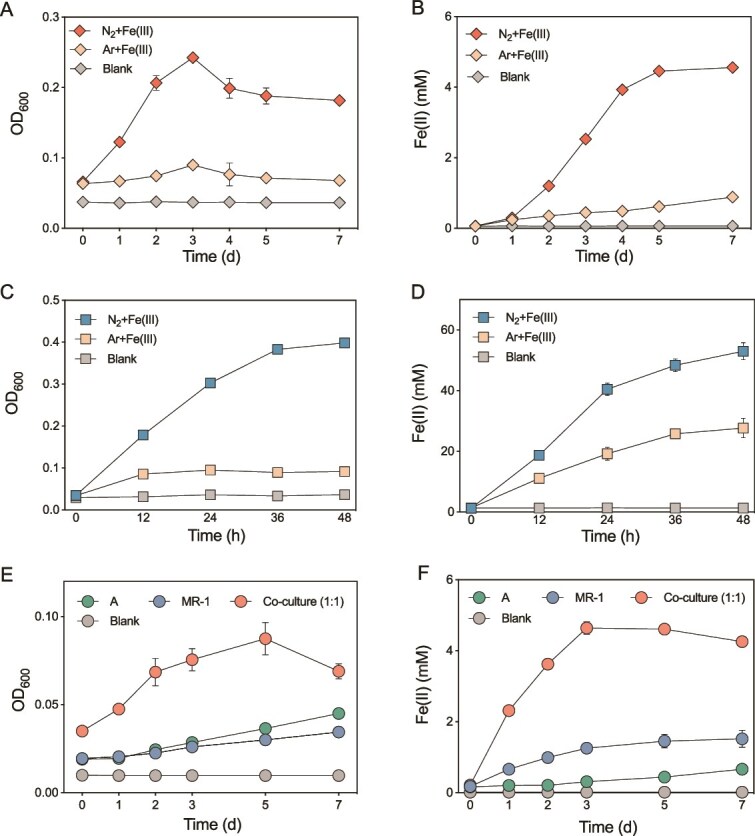
The effect of nitrogen fixation on growth and ferric iron reduction in pure culture experiments. (A) Growth curve and (B) ferric citrate reduction of *K. grimontii* N7 under N_2_ and Ar conditions. (C) Growth curve and (D) ferric citrate reduction of *G. sulfurreducens* PCA under N_2_ and Ar conditions. (E) Growth curve and (F) ferric citrate reduction of *A. humicireducens* SgZ-5T and *S. oneidensis* MR-1 in monocultures and co-culture under nitrogen-free conditions. The blank represents the abiotic control.

The intraspecies and interspecies effects of MNF on DIR were detected using batch pure cultures. The intraspecies results of two treatments, including N_2_ + Fe and Ar + Fe, showed that MNF (N_2_ group) significantly promoted the growth (OD_600_, increased by 200% and 344.4%, respectively) and DIR (maximum rate increased by 771.3% and 169.02%, respectively) of strains *K. grimontii* N7 (Fig. 1A and B) and *G. sulfurreducens* PCA (Fig. 1C and D). After 7 days of *K. grimontii* N7 incubation, nearly all 5 mM Fe(III) was reduced to Fe(II) in the N_2_ group. In contrast, the Ar group exhibited only a minor Fe(II) increase of 0.89 mM, indicating that Fe(III) reduction was negligible in the absence of N_2_ fixation (Fig. 1B). No significant increase in Fe(II) concentration was observed in the control group throughout the incubation. For *G. sulfurreducens* PCA, the total Fe(III) reduction under N_2_ reached 55.9 mM, 1.9 times greater than in the Ar group (Fig. 1D). These results collectively demonstrate that MNF is essential for promoting DIR in these strains, highlighting the critical role of MNF in microbial Fe(III) reduction metabolism.

For interspecies interactions, the model diazotroph *A. humicireducens* SgZ-5T and the model DIRB *S. oneidensis* MR-1 were co-cultured to assess the effect of MNF on DIR. Both individual strains and their co-culture exhibited distinct patterns of growth and Fe(III) reduction (Fig. 1E and F). Compared to monocultures, where *S. oneidensis* MR-1 and *A. humicireducens* SgZ-5T achieved only 30% and 14% Fe(III) reduction, respectively, the co-culture exhibited markedly enhanced performance, achieving complete reduction of 5 mM Fe(III) within 7 days. These results suggest that the presence of the diazotroph substantially promotes DIR by the DIRB.

### Effects of Fe(III) reduction on diazotrophic activity

**Figure 2:**
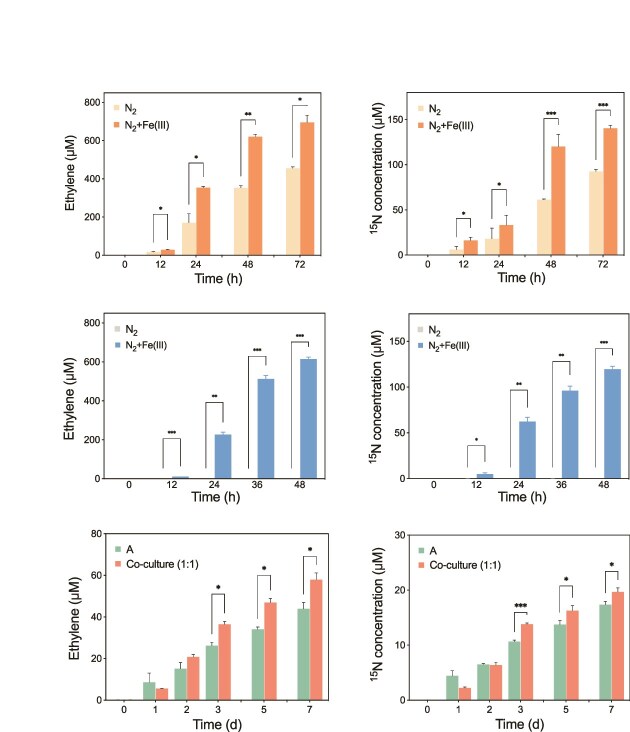
Nitrogen fixation activity in pure culture incubations. (A) Nitrogenase activity and (B) ^15^N fixation concentrations in *K. grimontii* N7 with or without ferric citrate. (C) Nitrogenase activity and (D) ^15^N fixation concentrations in *G. sulfurreducens* PCA with or without ferric citrate. (E) Nitrogenase activity and (F) ^15^N fixation in *A. humicireducens* SgZ-5T monoculture and co-culture. ^*^, ^**^, and ^***^ represent *P* <0.05, 0.01, and 0.001, respectively.

The effect of DIR on MNF within species was investigated using strains *K. grimontii* N7 and *G. sulfurreducens* PCA under conditions with or without Fe(III) amendment. Cultures amended with Fe(III)-citrate exhibited significantly higher nitrogenase activities (695.4 ± 37.9 μM) than those without Fe(III) reduction (455.5 ± 7.1 μM) after 3 days of incubation in strain N7 (Fig. 2A). Furthermore, the concentration of fixed ^15^N (140.2 ± 3.4 μM) was significantly higher in Fe(III)-amended cultures compared to those without Fe(III) (92.6 ± 2.3 μM) (Fig. 2B). Additionally, the total amino acid concentration (N-fixing products) in Fe(III)-amended cultures was ~1.5 times higher than in the nonamended treatments (Fig. S4). The nitrogenase activity of the respiratory diazotrophic DIRB *G*. *sulfurreducens* PCA was only observed when Fe(III)-citrate was added. After 48 h of incubation, strain PCA reduced ~614.6 μM of acetylene (Fig. 2C) and 59.8 μM of ^15^N_2_ (Fig. 2D) through MNF.

The interspecies effects of DIRB on diazotroph MNF during DIR were also explored. No nitrogenase activity was detected in the monoculture of *S. oneidensis* MR-1 (Fig. S5). However, the co-culture of *S. oneidensis* MR-1 and *A. humicireducens* SgZ-5T demonstrated enhanced N fixation activity, particularly during the middle and late stages, as indicated by the acetylene reduction and ^15^N isotope tracing (Fig. 2E and F). In contrast, no enhancement was observed in a co-culture lacking Fe(III) (Fig. S6), suggesting that DIR may play an important role in promoting MNF.

### Transcriptional responses of species to nitrogen fixation and Fe(III) reduction

**Figure 3:**
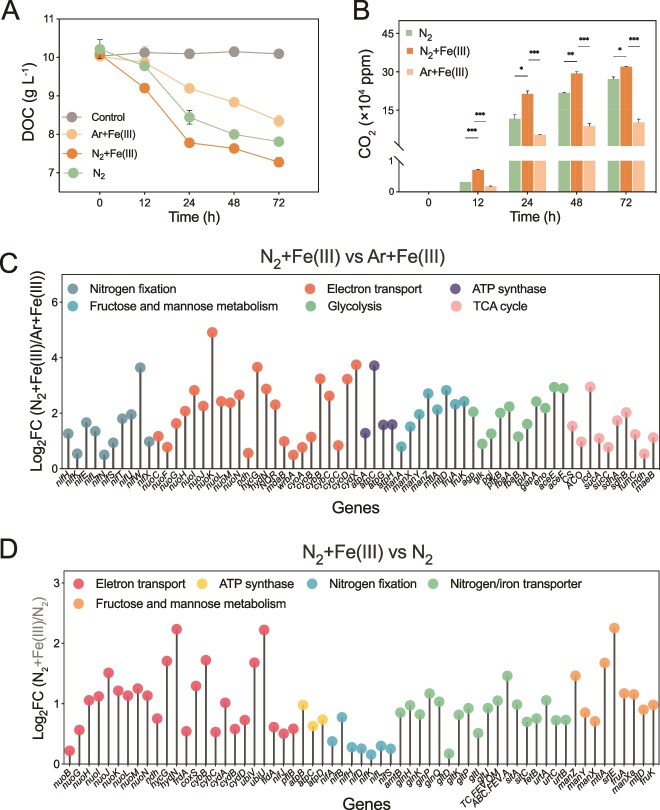
Characterization of the synergy between nitrogen fixation and Fe(III) reduction in intraspecific cultures. (A) CO_2_ production and (B) DOC consumption in strain *K. grimontii* N7 under different treatments. (C) Upregulated genes related to nitrogen fixation, electron transport, ATP synthase, and carbon metabolism in strain *K. grimontii* N7 under N_2_ versus Ar conditions. (D) Upregulated genes related to electron transport, ATP synthase, nitrogen fixation, and carbon metabolism in strain *K. grimontii* N7 with or without ferric iron addition. Gene expression across major pathways was measured by log_2_-fold change (Log_2_FC) under different treatments (*P* < 0.01). ^*^, ^**^, and ^***^ represent *P* < 0.05, 0.01, and 0.001, respectively.

To elucidate the mechanisms underlying the synergy between MNF and DIR within and between species, we conducted transcriptomic analyses under both intraspecific (*K. grimontii* N7) (Table S3) and interspecific (*A. humicireducens* SgZ-5T with *S. oneidensis* MR-1) conditions. In strain N7, a total of 908 genes were significantly upregulated under N_2_ + Fe(III) compared to the Ar + Fe(III) control (Fig. S7A). GO enrichment revealed that these DEGs were enriched in amino acid biosynthesis, ATP generation, carbohydrate metabolism, N compound transport, and nucleotide metabolism (Fig. S8A). KEGG annotation showed that *nifH* and N assimilation genes were upregulated by 3.6-fold and 2.7-fold, respectively (*P* < 0.01, Log_2_FC > 0.5) under N_2_ conditions (Fig. 3C and Fig. S9). Genes related to glycolysis, the tricarboxylic acid (TCA) cycle, NADH-quinone oxidoreductases (*nuo*), cytochrome oxidase (*cyo*), and ATP synthase (*atp*) were also upregulated by 2.9–4.9-fold during N fixation (Fig. 3C), consistent with increased CO_2_ production and DOC depletion (Fig. 3A and B). Under Fe(III)-reducing conditions, 520 genes were significantly upregulated, including those involved in NADH dehydrogenase, electron transfer, Fe-S cluster binding, and fermentation metabolism (*pflB* and *nifJ*), along with *atp* synthase components (*P* < 0.01, Log_2_FC > 0.5) (Fig. 3D and Figs. S7B, S8B). These transcriptional shifts coincided with enhanced C degradation, CO_2_ production, and ATP yield (Fig. 3A, B and Fig. S10A), indicating that Fe(III) reduction stimulates OM oxidation, providing energy and electrons to support N fixation. Collectively, these results confirm a metabolic feedback loop between MNF and DIR within species, where N fixation promotes C metabolism and biomass growth, which in turn enhances Fe(III) reduction.

**Figure 4:**
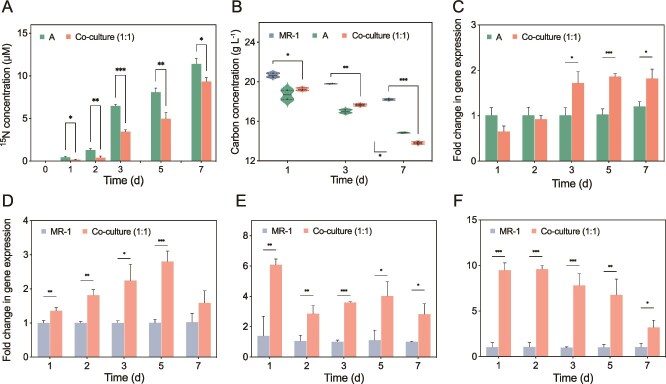
Characterization of the synergy between nitrogen fixation and Fe(III) reduction in interspecific cultures. (A) the concentration of extracellular ^15^N products in monoculture of *A. humicireducens* SgZ-5T and co-culture. (B) DOC (mannitol) consumption in *A. humicireducens* SgZ-5T and *S. oneidensis* MR-1 monocultures and co-culture. (C) Fold change in *nifH* gene expression in *A. humicireducens* SgZ-5T in co-culture compared to monoculture. The fold-change in gene transcription in *S. oneidensis* MR-1 in co-culture compared to monoculture: (D) *Shewanella* 16S rRNA gene, (E) *cymA* gene, and (F) *mtrA* gene. ^*^, ^**^, and ^***^ represent *P* < 0.05, 0.01, and 0.001, respectively.

**Figure 5:**
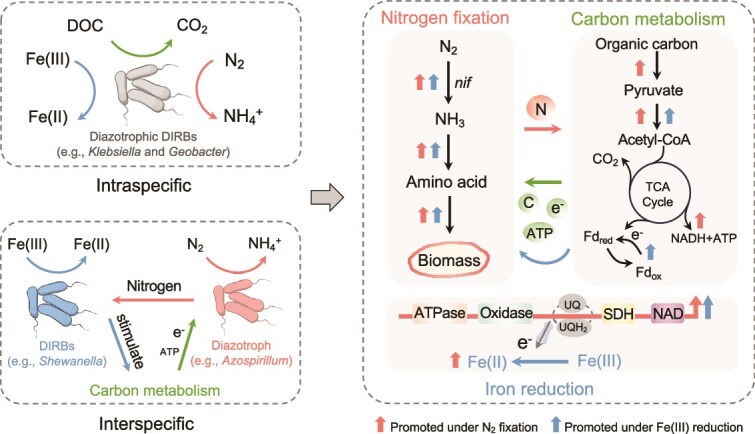
Schematic illustration of microbial nitrogen fixation and iron reduction synergy. Left: conceptual model illustrating the intraspecific and interspecific synergy between microbial nitrogen fixation (MNF) and dissimilatory iron reduction (DIR). Diazotrophic DIRBs (e.g. *K. grimontii* N7 and *G. sulfurreducens* PCA) are capable of simultaneously performing MNF and DIR. Iron-reducing bacteria (e.g. *S. oneidensis* MR-1) are distinguished from diazotrophs (e.g. *A. humicireducens* SgZ-5T). Right: the underlying mechanisms of MNF-DIR synergy. The three shaded areas represent microbial nitrogen fixation, carbon metabolism, and iron reduction, respectively. The diagram illustrates the mutual metabolic coupling, where nitrogen fixation supplies nitrogen to support carbon metabolism, biomass synthesis, and electron transfer, which in turn promotes Fe(III) reduction. As an electron acceptor, Fe(III) enhances the oxidation of organic matter and provides energy and electrons to sustain nitrogen fixation. Together, this synergy facilitates coupled nitrogen, iron, and carbon cycling.

A similar mutual reinforcement was observed in the co-culture of *A. humicireducens* SgZ-5T and *S. oneidensis* MR-1. Although DIRB *S. oneidensis* MR-1 lacks N-fixing capability, its 16S rRNA and electron transfer genes (*cymA* and *mtrA*) were significantly upregulated in co-culture (*P* < 0.05), accompanied by increased OM consumption and ATP production (Fig. 4B, D–F, and Fig. S10B). Transcription of the *nifH* gene in *A. humicireducens* SgZ-5T was also upregulated in the presence of *S. oneidensis* MR-1 (*P* < 0.05) (Fig. 4C), suggesting that DIR enhanced MNF through stimulated C oxidation and energy provision. The extracellular ^15^N concentration in co-cultures was significantly lower than that in monocultures (Fig. 4A), and amino acid secretion by *A. humicireducens* SgZ-5T was reduced from 378.72 μM in monoculture to lower levels in co-culture (*P* < 0.001) (Fig. S11), indicating that MR-1 likely utilized fixed N compounds for growth and DIR. These findings demonstrate that MNF and DIR are synergistic not only within single organisms but also across distinct species via nutrient and metabolic exchanges, thereby accelerating N, Fe, and C cycling (Fig. 5).

### Nitrogen fixation and Fe(III) reduction across diverse ecosystems

**Figure 6:**
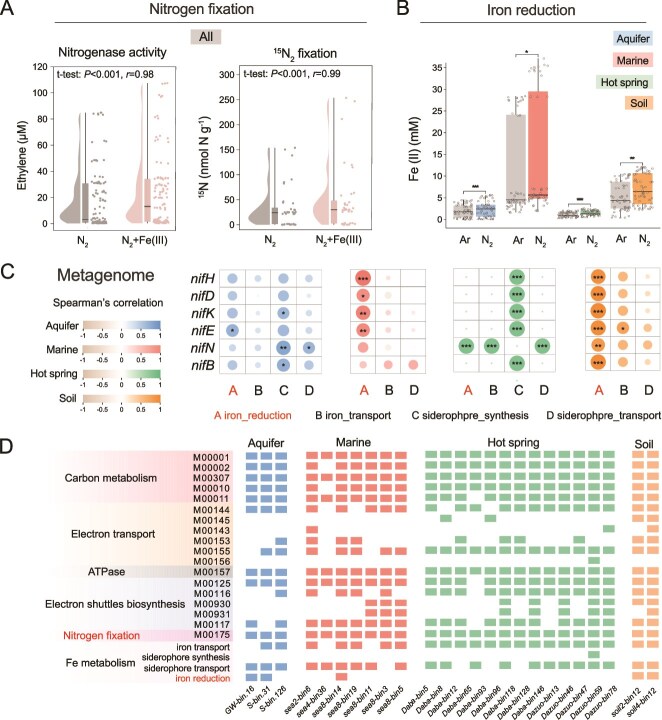
Synergistic interaction between microbial nitrogen fixation and Fe(III) reduction across diverse ecosystems. (A) Nitrogenase activity and ^15^N fixation and (B) Fe(III) reduction in microcosms from aquifer sediments, marine sediments, hot spring sediments, and soils. Different markers represent N_2_ and N_2_ + Fe(III) treatments, respectively. Box plots show Fe(II) production under Ar and N_2_ conditions. All experiments were conducted in triplicate (*n* = 3). (C) Spearman’s correlations between nitrogen fixation genes (e.g. *nifH*, *nifD*, and *nifK*) and dissimilatory iron reduction genes (e.g. *mtrA*, *mtrC*, *omcS*, and *omcZ*) across environmental metagenomes. (D) Metagenome-assembled genomes (MAGs) from various environments carrying both nitrogen fixation and Fe(III) reduction genes, indicating potential for functional coupling.

Microcosm experiments demonstrated that DIR significantly enhanced MNF in representative samples from aquifer waters/sediments (*P* < 0.001), marine sediments (*P* < 0.001), and soils (*P <* 0.001) (Fig. 6A and Figs. S12, S13). By the end of incubation, nitrogenase activities were significantly higher in Fe(III)-amended groups (1.1–8106.9 μM) than in the corresponding non-Fe(III) controls (0.3–884.9 μM) (Fig. S12). Consistent with these findings, ^15^N_2_ isotope tracing further confirmed the stimulatory effect of DIR on MNF across aquifer, marine, and soil microcosms (Table S6, Fig. 6A, and Fig. S13). Among the aquifer samples, LDS-20 exhibited the highest ^15^N fixation incorporation, with Fe(III) supplementation resulting in 237.3 nmol N g^−1^ of fixed ^15^N, significantly exceeding the 147.7 nmol N g^−1^ observed in the N_2_ control (*P* < 0.01). Similarly, Fe(III) addition increased ^15^N content in XHC and LDS-30 by 37.7% (*P* < 0.01) and 38.9% (*P* < 0.001), respectively (Fig. S13A). A comparable pattern was observed in marine sediments, where ^15^N fixation was significantly promoted by Fe(III) addition (*P* < 0.001). The fixed ^15^N concentrations reached 25.2 nmol, 61.3 nmol, and 3.12 nmol in A7–1, A7–7, and C4 samples, respectively (Fig. S13B). In soils, Fe(III) addition increased ^15^N fixation by 51.7%, 23.9%, and 21.1% in JH1, S07, and TSH samples, respectively (Fig. S13D). In contrast, Fe(III) addition did not enhance nitrogenase activity and ^15^N fixation in hot spring sediments (Figs. S12C, S13C). Across all ecosystems, Fe(III) reduction was consistently stimulated under N_2_ incubation compared to Ar controls (Fig. 6B and Fig. S14). In marine and hot spring sediments, DIR was accelerated during the early incubation phase, with Fe(II) concentrations increasing by 15.5%–40.6% (*P <* 0.05) (Fig. S14B and C). In aquifer sediments and soils, Fe(III) reduction peaked during mid-incubation, with Fe(II) yields elevated by 14.8%–38.2% (*P <* 0.05) (Figs. S14A, D). These results collectively demonstrate that the synergistic interaction between MNF and DIR occurs in different ecosystems.

To further elucidate the underlying metabolic potentials, metagenomic and metatranscriptomic analyses were conducted on representative samples from aquifer waters/sediments, marine sediments, hot spring sediments, and soils. Functional genes associated with MNF (e.g. *nifHDKENB*) and DIR (e.g. *omc*, *cym*, and *mtr*) were detected at both DNA and RNA levels. The abundance and transcription of nitrogenase genes were significantly positively correlated with genes encoding multiheme c-type cytochromes (MHCs), including *mtrABC* and *omcSZ*, across all ecosystems (*P* < 0.05) (Fig. 6C and Figs. S15, S16). Moreover, *nif* gene clusters also exhibited strong correlation with genes involved in N transport (*amt*), N assimilation (*glt*, *gln*, and *gdh*), electron transport (*nuo*, *cyd*, *fix*, and *kor*), F-type ATPases (*atp*), and Fe transport (*feo*) (*P* < 0.05) (Figs. S15 and S16). Metagenome-assembled genomes (MAGs) of medium quality (completeness >50%, contamination <10%) containing key genes for both N fixation (*nif*) and DIR (MHCs: *omc*, *cym*, and *mtr*) were retrieved from aquifer, soil, and marine environments. These included MAGs such as GW-bins 16 (*Nitrospirota*), S-bins 31 (*Thermoplasmatota*), soil2-bins 12 (*Archaea*), soil4-bins 12 (*Thermoplasmatota*), and sea8-bins 14 (*Bacteria*) (Fig. 6D, Fig. S17, and Table S7). Although diazotrophic genomes recovered from hot spring sediments lacked canonical MHCs, they contained genes involved in electron transport and redox cofactor biosynthesis, such as riboflavin and menaquinone pathways and quinone oxidoreductase enzymes. KEGG Module analysis further confirmed the presence of genes related to central C metabolism (e.g. glycolysis, pyruvate oxidation, and citrate cycle), electron transport (e.g. NADH dehydrogenase, quinone oxidoreductase, cytochrome c oxidase), ATP synthesis (F-type ATPase), electron shuttle biosynthesis (riboflavin/menaquinone), N fixation (*nif*), and DIR (*omc*, *cym*, and *mtr*) across MAGs from aquifer, marine, hot springs, and soils environments (Fig. 6D). These findings demonstrate that microbial communities across diverse ecosystems possess the metabolic potential to support the synergy of MNF and DIR.

## Discussion

### Evidence and mechanisms underlying the synergy between microbial nitrogen fixation and iron reduction

Previous studies have speculated on potential correlations between diazotrophs and DIRBs in various ecosystems, including soils [49], coastal sediments [12], and wetlands [50]. Our recent research expanded this understanding to groundwater systems, revealing positive MNF-DIR interactions in previously unexplored environments [14]. However, these earlier investigations primarily relied on community composition and correlation-based analyses, lacking direct evidence. In this study, we provide integrated evidence from intraspecies, interspecies, and environmental microcosms, demonstrating a consistent and reciprocal synergy between MNF-DIR.

Under N_2_ fixation conditions, both fermentative and respiratory diazotrophic DIRBs exhibited accelerated growth and Fe(III) reduction (Fig. 1), and the presence of Fe(III) substantially enhanced MNF, confirming a mutualistic relationship (Fig. 2). At the intraspecific level, transcriptomic data revealed that MNF significantly upregulated multiple C metabolism pathways, including glycolysis, the TCA cycle, and electron transport chain components (Fig. 3C). This enhanced C oxidation increased biomass production, thereby fueling Fe(III) reduction. In turn, Fe(III) reduction reinforced MNF by improving the energetic balance of the cell: in fermentative strains, Fe(III) acted as a secondary electron acceptor during organic substrate fermentation, increasing energy yield [51, 52] (Figs. 2A, B and 3A, D), whereas in respiratory strains, Fe(III) served directly as a terminal electron acceptor, accelerating electron transfer and ATP synthesis [51, 53, 54], which supported N_2_ fixation (Fig. 2C and D). At the interspecific level, co-culture assays revealed a bidirectional exchange of resources between diazotrophs and nondiazotrophic DIRBs. Nondiazotrophic DIRBs indirectly promoted N_2_ fixation by sustaining Fe(III)-reducing respiration, which facilitated C oxidation and electron flow in the shared environment (Fig. 4B, E, and  F). Conversely, diazotrophs provided fixed N as an essential nutrient, thereby stimulating DIRB growth and strengthening their Fe(III)-reducing capacity (Fig. 4A, D). Microcosm experiments quantified this synergy, estimating that MNF increased Fe(II) accumulation by 22.6–31.9% in aquifer, marine, hot spring, and soil environments (Fig. S14), and the reduction of 1 mmol Fe(III) could promote 11.54–448.47 μmol of N fixation (Table S8). Collectively, these findings, together with strong cross-ecosystem correlations among genes involved in nitrogenase activity, C metabolism, and DIR (Fig. 6 and Figs. S15, S16), jointly indicate a tightly integrated metabolic network linking MNF, DIR, and C turnover (Fig. 5).

The coupling between MNF, DIR, and C metabolism may help explain the widespread occurrence of MNF in eutrophic environments. In soils, OM inputs from roots and microbial processes directly promote N-C interactions [55], which could reinforce the synergy between MNF and DIR. In marine environments, particulate OM serves as a key C source for heterotrophic diazotrophs and DIRBs. Within sinking particles, microscale N depletion creates niches favorable to diazotrophs [22, 56], and high particle flux in such waters sustains substrate input, making bottom sediments and particle-rich zones potential hotspots for MNF-DIR interactions. In groundwater systems, OM is often derived from sediments, peat layers, or surface recharge [57, 58]. Hydrological gradients between recharge and discharge areas influence redox conditions and microbial metabolism [14, 59], and MNF-DIR coupling is more likely in discharge zones where OM concentration and active diazotrophic DIRB biomass are relatively high [14]. Compared to soils and marine sediments, OM scarcity and prevailing redox conditions [59] may reduce the magnitude of MNF-DIR interactions in aquifers, consistent with weaker gene correlations in our metagenomic datasets. In extreme oligotrophic habitats such as hot springs, C-fixing autotrophs and algae residues are the main C sources for heterotrophs (our unpublished data and previously published data [60]). Although these inputs can enable MNF-DIR interactions, the dominance of autotrophs, low OM availability, and reduced DIRB activity likely constrain Fe(III) as an energy source for N fixation [61], resulting in weaker synergy (hot spring sediments in Fig. 6c and Fig. S15C). Overall, although the mechanistic basis of MNF-DIR synergy is consistent across ecosystems, the location and intensity of activity hotspots are likely shaped by OM supply pathways, redox dynamics, and microbial community composition.

### Environmental implications of microbial nitrogen fixation-Fe(III) reduction synergy

Anthropogenic activities, particularly the excessive application of N-based fertilizers in agricultural soils, have led to the accumulation of reactive N in subsurface environments, contributing to widespread water pollution [62, 63]. Our findings suggest that the synergy between MNF and DIR may serve as a natural pathway to enhance N and Fe pools in soils. In terrestrial ecosystems, such synergy may help maintain N supply for plant uptake [64] and stimulate soil organic carbon (SOC) turnover and microbial productivity [65, 66]. By sustaining *in situ* generation of bioavailable N, MNF-DIR interactions have the potential to limit excessive fertilizer application, thereby reducing the leaching of reactive N into aquifers, lakes, and estuaries.

Such microbial interactions are further supported by Fe redox cycling, particularly under fluctuating oxygen conditions. In these settings, Fe(III) reduction by DIRBs and subsequent oxidation of Fe(II) by nitrate-reducing or oxygen-dependent Fe(II)-oxidizers closely couple Fe cycling to OM transformation [67–69]. This cyclical process may continuously fuel MNF, and N fixation enhances microbial growth and Fe cycling, forming a self-reinforcing biogeochemical loop. Although the capacity of MNF-DIR synergy to mitigate N pollution has yet to be quantitatively evaluated, its potential to buffer reactive N fluxes through natural microbial activity is promising. MNF-induced DIR may also influence Fe mineral transformation dynamics, with potential implications for the mobilization or immobilization of trace elements such as As [70, 71]. In marine systems, coupled N-Fe-C cycling has also been shown to promote primary productivity and nutrient fluxes by enhancing diazotrophic and DIRB activity [67, 72].

Beyond nutrient cycling, MNF-DIR interactions may also regulate greenhouse gas emissions. Coupled MNF and DIR have been linked to altered CH_4_ emissions [73], and recent research has proposed N_2_O fixation by diazotrophs as an alternative N_2_O sink, potentially accounting for up to 60% of total N_2_O reduction in the Pacific Ocean [74, 75]. Our pure culture experiments further support this, showing that DIR enhances both N_2_O fixation and DOC consumption, thereby promoting the growth of diazotrophic DIRB (Fig. S18). These findings highlight the potential of DIR-driven MNF in reducing N_2_O emissions in natural and agricultural systems.

In summary, the synergy between MNF and DIR is significant in nutrient cycling and greenhouse gas regulation. This study offers insights into the metabolic and ecological linkages between N, Fe, and C cycles, underscoring the need for future studies to assess the long-term impacts and practical applications of this microbial interaction in sustainable agriculture and environmental remediation.

## Supplementary Material

Supplementary_materials_wraf212

## Data Availability

Raw RNA-Seq data have been deposited in the NCBI database (accession number: PRJNA1172356). Metagenomic sequencing reads from the aquifer and hot spring samples are available from our previous study under NCBI BioProject accession numbers PRJNA882225 and PRJNA943127. Metatranscriptomic sequencing reads from the aquifer, marine sediments, and soils are available under NCBI BioProject accession numbers PRJNA884812 and PRJNA1253882.
