# Supplementary material for: Synergistic interaction between microbial nitrogen fixation and iron reduction in the environment
Source: ISME J. 2025 Sep 23;19(1):wraf212. doi: 10.1093/ismejo/wraf212 (PMC12516963; doi:10.1093/ismejo/wraf212)
Supplement: Supplementary_materials_wraf212 [file supplementary_materials_wraf212.docx]

Supplementary materials

**Synergistic interaction between microbial nitrogen fixation and iron reduction in the environment**

Xiaohan Liu ^1, 2^, Ping Li ^1, 2 *^, Keman Bao ^1, 2^, Yaqi Wang ^1, 2^, Helin Wang ^1, 2^, Yanhong Wang ^1, 2^, Zhou Jiang ^1^, Yi Yang^3^, Songhu Yuan^1, 2^, Andreas Kappler^4, 5^, Yanxin Wang^1, 2^

^1^ State Key Laboratory of Geomicrobiology and Environmental Changes, China University of Geosciences, Wuhan, 430074, P.R. China

^2^ Hubei Key Laboratory of Yangtze Catchment Environmental Aquatic Science, School of Environmental Studies, China University of Geosciences, Wuhan, 430074, P.R. China

^3^ College of Marine Science and Technology, China University of Geosciences, Wuhan, 430074, P.R. China

^4^ Department of Geosciences, University of Tübingen, Tübingen, Germany.

^5^ Cluster of Excellence: EXC 2124: Controlling Microbes to Fight Infection, Tübingen, Germany.

^*^Corresponding author: Ping Li, pli@cug.edu.cn

**Contents of this file**

Tables 8

Figures 18

**Section S1: FWAFC medium for *Geobacter sulfurreducens* PCA**

The preparation process of the medium was as follows:

First, ferric citrate was placed in a large beaker, heated until dissolved, and then mixed with pre-cooled ultrapure water. The pH was adjusted to approximately 6.0 using sodium hydroxide. Subsequently, 50 mL of Ca/Mg Mix, 10 mL of Trace Mineral Mix, and 1 mL of Wolfe’s Vitamin Solution (ELITE-MEDIA, Germany) were added sequentially and mixed thoroughly. Next, 1.64 g of sodium acetate was weighed and fully dissolved with stirring, and the pH was readjusted to approximately 7.0 using a sodium hydroxide solution. The prepared solution was then transferred to a volumetric flask and adjusted to a final volume of 1 L. Subsequently, 2.0 g of sodium bicarbonate was added and stirred until completely dissolved. The medium was aliquoted into 60 mL serum bottles and deoxygenated by purging with a gas mixture (N_2_ : CO_2_ = 80 : 20 or Ar : CO_2_ = 80 : 20). Finally, the medium was sterilized at 121°C for 20 min and stored for later use. The compositions of the Ca/Mg Mix and Trace Mineral Mix stock solutions are detailed in Tables R1 and R2.

Table R1 Ca/Mg Mix composition

| Component | Concentration (g L^-1^) |
| --- | --- |
| CaCl_2_·2H_2_O | 2 |
| MgSO_4_·7H_2_O | 10 |

Table R2 Trace Mineral Mix composition

| Component | Concentration (g L^-1^) |
| --- | --- |
| Nitrilotriacetic acid (NTA) | 1.5 |
| MnCl_2_·4H_2_O | 0.1 |
| Fe_2_(SO_4_)_3_·7H_2_O | 0.5 |
| CoCl_2_·6H_2_O | 0.17 |
| ZnCl_2_ | 0.1 |
| CuSO_4_·5H_2_O | 0.03 |
| KAl(SO_4_)_2_·12H_2_O | 0.005 |
| H_3_BO_3_ | 0.005 |
| Na_2_MoO_4_ | 0.09 |
| NiCl_2_ | 0.05 |
| Na_2_WO_4_·2H_2_O | 0.02 |
| Na_2_SeO_4_ | 0.1 |

**Section S2: N_2_O assimilation experiments in strain *Klebsiella grimontii* N7**

Biological N_2_O assimilation was validated through isotopic tracing experiments using doubly ^15^N-labeled ^46^N_2_O as a substrate. The increase in ^15^N concentration in biomass was measured to confirm N_2_O fixation. High purity of ^46^N_2_O was prepared using the “denitrifier method” described by Sigman Sigman et al (2001), in which ^15^N-labeled sodium nitrate was converted to ^46^N_2_O by the bacterium *Pseudomonas aureofaciens* (ATCC# 13985). In the experiment investigating the interaction between N_2_O fixation and Fe(III) reduction, three experimental groups were set up: B-N_2_O, Fe-N_2_O, Fe-Ar, and a non-biological control group (Control). In the B-N_2_O and Fe-N_2_O groups, 10% of the headspace volume (6 mL) was supplemented with ^46^N_2_O as the nitrogen source, while 0.2 mM ferric citrate was added to the Fe-N_2_O and Fe-Ar groups to examine the influence of Fe(III) reduction on N_2_O fixation. Each experimental group was conducted in replicate. All samples were incubated at 30°C, in the dark, and shaken at 150 rpm. At the end of incubation, samples for ^15^N measurement were preserved in 12.5 mL vials (Labco Exetainer, Lampeter, UK), with 0.1 mL of 50% w/v ZnCl_2_ added as a preservative.

**Section S3: RNA sampling for transcriptomics**

Due to the use of a nitrogen-free medium, the biomass of *Klebsiella grimontii* N7 was relatively low during the early cultivation phase (0–12 h) (Fig. R4A). Therefore, we collected RNA samples at 8, 12, 24, 48, and 72 hours to ensure sufficient biomass for transcriptomic analysis. Our results indicate that *nifH* gene expression (%) peaked at 12 h (Fig. R4F), making it the most appropriate time point for analyzing nitrogenase transcription, despite not coinciding with the highest nitrogenase activity (Fig. R4C) or the logarithmic growth phase (Fig. R4A). Since gene expression typically precedes enzymatic function, we selected 12 h as the optimal time point for transcriptomic analysis to best capture the regulatory mechanisms underlying nitrogen fixation.

**
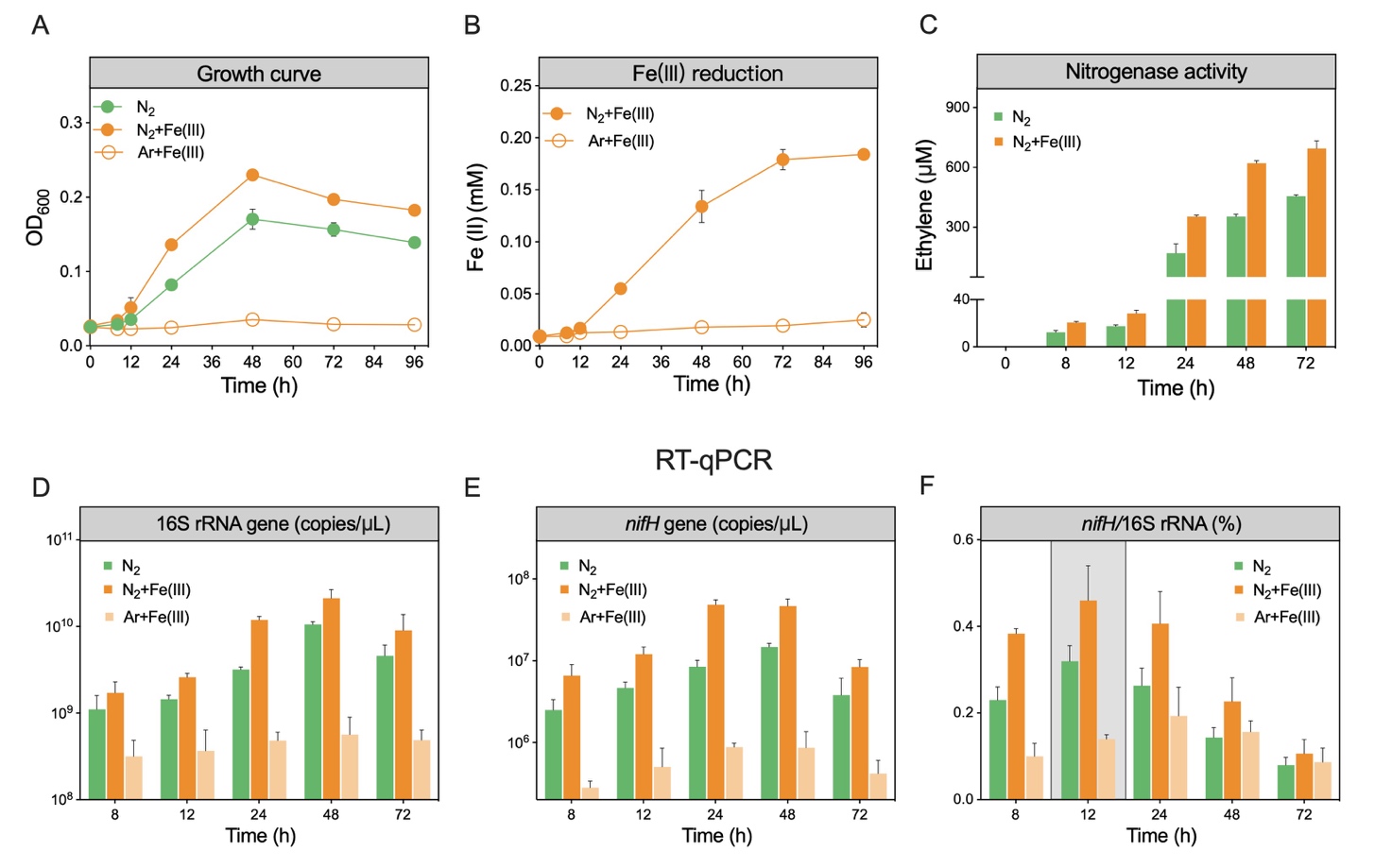
**

**Figure R1** Physiological activity and gene expression of strain *Klebsiella grimontii* N7 in transcriptomic analysis. (A) Growth curve; (B)Iron reduction; (C) Nitrogenase reduction; (D) 16S rRNA gene expression; (E) *nifH* gene expression; (F) the relative abundance of *nifH* gene (%). The gray shadows in Figure E represent sampling time points for the transcriptome.

**Section S4: Metagenomic and metatranscriptomic sequencing**

DNA and RNA were extracted from environmental samples using the FastDNA SPIN (MP Biomedicals, Santa Ana, CA, USA) and RNeasy PowerSoil Total RNA (Qiagen, Hilden, German) kits, respectively, following the manufacturer’s protocols. The quality and quantity of DNA/RNA were examined using a NanoDrop-2000 spectrophotometer (Thermo Fisher Scientific, Waltham, MA, USA) and agarose (1%) (Biowest, Riverside, MO, USA) gel electrophoresis. All DNA and RNA samples were stored at −80°C for quantitative and sequencing analysis.

Total DNA and RNA were sent to Magigene Technology (Guangzhou, China) as soon as possible for metagenomic and metatranscriptomic sequencing using a HiSeq platform (Illumina). Metagenomic and metatranscriptomic sequencing raw data were trimmed to produce clean data using Trimmomatic (Version 0.36) [2] and de novo assembled individually to contigs using metaSPAdes [3] and Trinity [4], respectively. Raw reads generated after metatranscriptomic sequencing were filtered using SortMeRna to remove tRNA and rRNA sequences before assembly [5]. The contigs with <500 bp were omitted. The open reading frames (ORFs) on contigs were predicted using Prodigal’s metagenome mode (v2.6.3) [6] and quantified using Salmon (v1.3.0) [7]. The ORFs were annotated by HMMSEARCH (v3.3.2) against KOfam (a customized Hidden Markov model (HMM) database of KEGG Orthologs) (-e 10^-5^) [8]. Functional annotation was performed using KEGG Orthologs (KO) and KEGG Module databases to characterize the relative abundance of nitrogenases and their biogeochemical networks. FeGenie was used to search for ORFs encoding Fe-reducing genes [9].

Metagenomic binning: Binning was conducted using metaBAT2 and MaxBIN2 by MetaWRAP (v1.3) pipeline with default parameters [10]. The resulting bins were further analyzed using CheckM (1.2.0), and medium-quality MAGs with completeness > 50% and contamination < 10% were kept for downstream analysis [11,12]. The classify_wf and de_novo_wf modules of the Genome Taxonomy Database Toolkit (GTDB-Tk v2.4.0) were used to classify the taxonomy of MAGs [13]. The functional gene annotation for each medium-quality MAG was conducted using the MetaWRAP pipeline with the default parameters [10]. In addition, FeGenie was used to search for ORFs encoding Fe-reducing genes [9].

**Table S1** Geochemical parameters of sediment/soil samples from different ecosystems used in microcosms.

|  | Samples | Location | Depth (m) | ORP (mv) | DO  (mg L^-1^) | Salinity | Temp (°C) | Fe  (mmol L^-1^) |
| --- | --- | --- | --- | --- | --- | --- | --- | --- |
| Aquifer sediment | X2 | 41.02 N,106.94 E | 20 | -86.4 | / | / | 12.2 | 0.94 |
|  | L2 | 40.96 N,107.00 E | 20 | -221.2 | / | / | 11.8 | 5.90 |
|  | L3 | 40.96 N,107.00 E | 30 | -194.2 | / | / | 12.7 | 3.05 |
| Soil | JH1 | 31.14 N,112.37 E | 0-0.1 | 197.5 | / | / | 17.3 | 7.82 |
|  | S07 | 30.31 N,113.82 E | 0-0.1 | / | / | / | 11.0 | 12.67 |
|  | TSH | 30.30 N,113.89 E | 0-0.1 | / | / | / | 15.7 | 12.09 |
| Hot spring sediment | DB-A-1 | 30.52 N,98.06 E | 0-0.1 | -301 | / | / | 63 | 2.12 |
|  | DB-A-3 |  | 0-0.1 | -121 | / | / | 54 | 1.19 |
|  | DB-B-1 |  | 0-0.1 | -298 | / | / | 61 | 7.79 |
|  | DB-B-3 |  | 0-0.1 | -91 | / | / | 54 | 3.25 |
| Marine sediment | A7-1 | 30.5 N,122.2 E | 10.2 | / | 3.6 | 19.2 | 26.4 | 10.76 |
|  | A7-7 | 29.7 N,123.8 E | 72 | / | 4.5 | 32.9 | 28.0 | 6.05 |
|  | C4 | 31.2 N,121.8 E | 11.2 | / | 4.1 | 0.1 | 28.6 | 34.10 |

**Table S2** Primer pairs used in this study and correspondence RT-qPCR protocols.

| Target gene | Primer pair | | | Sequences | Reaction conditions | | | Methods |  |
| --- | --- | --- | --- | --- | --- | --- | --- | --- | --- |
| Bacterial  16S rRNA | | 27F  1492R | 5´- AGAGTTTGGATCMTGGCTCAG -3´  5´- CGGTTACCTTGTTACGACTT -3 | | | 95°C for 5 min; 35 cycles of 30 s at 94°C, 30 s at 55°C, 2 min at 72°C, and 7 min at 72°C. | PCR | | |
| Bacterial  16S rRNA  (V4 region) | | 515F  806R | 5´- GTGCCAGCMGCCGCGGTAA -3´  5′- GGACTACHVGGGTWTCTAAT -3′ | | | 94°C for 3 min, 35 cycles of 45 s at 94°C, 60 s at 50°C, 90s at 72°C and 10 min at 72°C. | qPCR | | |
| *nifH* | | PolF  PolR | 5´- TGCGAYCCSAARGCBGACTC -3´  5´- ATSGCCATCATYTCRCCGGA -3 | | | 95°C for 10 min, 40 cycles of 95°C for 15s, 56°C for 45 s, and 72°C for 45 s. | qPCR | | |
| *Shewanella*  16S rRNA | | 640F  815R | 5’-RACTAGAGTC TTGTAGAGG-3’  5’-AAGDYACCA AAYTCCGAGT-3’ | | | 95°C for 3 min; 40 cycles of 15 s at 95°C, 30 s at 55°C, and 30 s at 72°C. | qPCR | | |
| *Shewanella*  *cymA* | | CymA-F  CymA-R | 5’-GATGCGTTCTGTATGTCTTG-3’  5’-ACGGAAGTAAGCCAATGC-3’ | | | 95°C for 30 s; 40 cycles of 5 s at 95°C, and 60 s at 60°C. | qPCR | | |
| *Shewanella*  *mtrA* | | MtrA-F  MtrA-R | 5’-AGCGTGAATGATACCTGTTA-3’  5’-ATGAACCTGACTATGGCAAT-3’ | | | 95°C for 30 s; 40 cycles of 5 s at 95°C, and 60 s at 60°C. | qPCR | | |

- PCR of 16S rRNA genes was performed in a total volume of 25 μL containing 2.5 μL PrimeScript buffer, 1 μL dNTP Mixture, 0.3 μL TaKaRa Ex Taq, 18.2 μL dd H_2_O, 1 μL of each primer, and 1μL DNA or RNA sample.
- qPCR was performed in a total volume of 15 μL containing 7.5 μL TB Green Premix Ex Taq Ⅱ, 1 μL forward and reverse primers, 0.3 μL ROX Reference Dye Ⅱ, 1 μL diluted template, and 4.2 μL RNase-free water.

**Table S3** Summary of transcriptome sequencing data and transcriptome assembly of strain *Klebsiella* *grimontii* N7.

| Sample Name | Raw reads | Raw Q20  (%) | Raw Q30  (%) | Clean Reads | Clean Q20  (%) | Clean Q30  (%) |
| --- | --- | --- | --- | --- | --- | --- |
| Burk | 10,938,523 | 92.59 | 81.12 | 10,399,996 | 94.87 | 85.19 |
| Burk | 11,773,693 | 92.61 | 81.03 | 11,186,067 | 94.89 | 85.13 |
| Burk | 10,851,900 | 92.47 | 80.53 | 10,272,038 | 94.80 | 84.78 |
| Burk+Fe | 11,144,068 | 92.50 | 80.89 | 10,558,610 | 94.89 | 85.18 |
| Burk+Fe | 11,047,853 | 92.46 | 80.77 | 10,490,580 | 94.81 | 85.02 |
| Burk+Fe | 10,236,657 | 91.66 | 78.79 | 9,742,101 | 93.99 | 82.95 |
| Burk+Fe+Ar | 8,683,616 | 97.12 | 92.35 | 8,437,994 | 97.99 | 93.61 |
| Burk+Fe+Ar | 8,490,047 | 97.18 | 92.56 | 8,257,889 | 98.07 | 93.83 |
| Burk+Fe+Ar | 8,167,003 | 97.21 | 92.51 | 7,928,530 | 98.09 | 93.77 |

**Table S4** Summary statistics for the assembly of metagenomic sequencing from various environmental samples.

|  | Sample | Trimmed reads | Number of contigs | Contig N50 |
| --- | --- | --- | --- | --- |
| Groundwater | G1 | 2.44E+07 | 205,929 | 1,161 |
|  | G2 | 4.19E+07 | 561,917 | 1,001 |
|  | G27 | 2.97E+07 | 364,855 | 873 |
|  | G30 | 3.78E+07 | 470,596 | 844 |
| Aquifer sediment | X2 | 3.00E+07 | 388,016 | 834 |
|  | X4 | 3.91E+07 | 502,033 | 1,007 |
|  | X6 | 2.36E+07 | 303,798 | 851 |
|  | L2 | 3.80E+07 | 443,302 | 1,567 |
|  | L4 | 3.22E+07 | 158,153 | 1,567 |
|  | L6 | 4.32E+07 | 601,614 | 748 |
| Hot spring | DB | 6.52E+07 | 830,879 | 2,362 |
|  | DZ | 6.24E+07 | 222,303 | 3,551 |
|  | ZM | 4.82E+07 | 46,849 | 21,399 |
| Soil | JH1 | 4.75E+07 | 712,382 | 657 |
|  | S07 | 3.16E+07 | 495,797 | 854 |
|  | TSH | 3.56E+07 | 445,844 | 732 |
|  | S1 | 5.03E+07 | 875,960 | 693 |
|  | S2 | 5.69E+07 | 868,266 | 698 |
|  | S3 | 5.09E+07 | 715,607 | 688 |
|  | S4 | 5.22E+07 | 1,068,941 | 762 |
|  | S5 | 5.07E+07 | 1,054,347 | 756 |
|  | S6 | 2.19E+07 | 395,549 | 623 |
|  | S7 | 3.34E+07 | 630,252 | 735 |
| Marine | A7-1 | 5.00E+07 | 843,733 | 834 |
|  | A7-7 | 6.47E+07 | 542,113 | 706 |
|  | C4 | 5.96E+07 | 876,834 | 931 |
|  | M1 | 4.12E+07 | 254,880 | 2,022 |
|  | M2 | 3.67E+07 | 442,442 | 1,153 |
|  | M3 | 4.50E+07 | 773,162 | 1,115 |
|  | M4 | 4.14E+07 | 628,003 | 1,271 |
|  | M5 | 5.45E+07 | 737,986 | 692 |
|  | M6 | 3.90E+07 | 485,928 | 1,279 |
|  | M7 | 4.85E+07 | 589,621 | 698 |
|  | M8 | 5.15E+07 | 914,253 | 754 |

**Table S5** Summary statistics for the assembly of metatranscriptomic sequencing from various environmental samples.

|  | Sample | Trimmed reads | Number of contigs | Contig N50 |
| --- | --- | --- | --- | --- |
| Groundwater | G1 | 2.45E+07 | 346,167 | 858 |
|  | G2 | 2.32E+07 | 400,011 | 816 |
|  | G27 | 2.58E+07 | 220,056 | 2,136 |
|  | G30 | 3.38E+07 | 165,891 | 1,178 |
| Aquifer sediment | X2 | 2.78E+07 | 495,982 | 842 |
|  | X4 | 2.96E+07 | 536,012 | 948 |
|  | X6 | 2.75E+07 | 321,885 | 1,035 |
|  | L2 | 1.90E+07 | 360,058 | 802 |
|  | L4 | 2.38E+07 | 341,827 | 867 |
|  | L6 | 3.05E+07 | 168,643 | 1,162 |
| Soil | JH1 | 4.20E+07 | 92,403 | 698 |
|  | S07 | 3.42E+07 | 106,441 | 856 |
|  | TSH | 3.42E+07 | 43,961 | 800 |
| Marine | A7-1 | 3.31E+07 | 70,265 | 1,440 |
|  | A7-7 | 5.39E+07 | 21,294 | 910 |
|  | C4 | 4.23E+07 | 46,084 | 1,677 |

**Table S6** Nitrogen isotopic abundance and total nitrogen content of environmental samples incubated with ^15^N_2_ for eight days.

|  | Sample | Treatment | | | ^15^N atom% excess sample | TN (%) |
| --- | --- | --- | --- | --- | --- | --- |
| Marine sediment | A7-1 | | Burk | 5.02 | | 0.061 |
|  | A7-1 | | Burk | 4.85 | | 0.063 |
|  | A7-1 | | Burk | 4.82 | | 0.063 |
|  | A7-1 | | Burk+Fe(III) | 5.46 | | 0.066 |
|  | A7-1 | | Burk+Fe(III) | 5.81 | | 0.064 |
|  | A7-1 | | Burk+Fe(III) | 5.86 | | 0.065 |
|  | A7-7 | | Burk | 5.21 | | 0.115 |
|  | A7-7 | | Burk | 5.76 | | 0.113 |
|  | A7-7 | | Burk | 5.44 | | 0.114 |
|  | A7-7 | | Burk+Fe(III) | 7.47 | | 0.109 |
|  | A7-7 | | Burk+Fe(III) | 7.63 | | 0.113 |
|  | A7-7 | | Burk+Fe(III) | 7.69 | | 0.116 |
|  | C4 | | Burk | 0.09 | | 0.108 |
|  | C4 | | Burk | 0.12 | | 0.112 |
|  | C4 | | Burk | 0.10 | | 0.109 |
|  | C4 | | Burk+Fe(III) | 0.38 | | 0.108 |
|  | C4 | | Burk+Fe(III) | 0.41 | | 0.107 |
|  | C4 | | Burk+Fe(III) | 0.42 | | 0.108 |
| Soil | S07 | | Burk | 1.61 | | 0.216 |
|  | S07 | | Burk | 1.61 | | 0.211 |
|  | S07 | | Burk | 1.61 | | 0.219 |
|  | S07 | | Burk+Fe(III) | 1.95 | | 0.211 |
|  | S07 | | Burk+Fe(III) | 1.96 | | 0.212 |
|  | S07 | | Burk+Fe(III) | 2.14 | | 0.216 |
|  | TSH | | Burk | 3.60 | | 0.100 |
|  | TSH | | Burk | 3.60 | | 0.099 |
|  | TSH | | Burk | 3.56 | | 0.096 |
|  | TSH | | Burk+Fe(III) | 3.96 | | 0.105 |
|  | TSH | | Burk+Fe(III) | 4.01 | | 0.105 |
|  | TSH | | Burk+Fe(III) | 4.12 | | 0.107 |
|  | JH1 | | Burk | 3.68 | | 0.096 |
|  | JH1 | | Burk | 4.17 | | 0.079 |
|  | JH1 | | Burk | 3.72 | | 0.081 |
|  | JH1 | | Burk+Fe(III) | 4.32 | | 0.098 |
|  | JH1 | | Burk+Fe(III) | 5.14 | | 0.092 |
|  | JH1 | | Burk+Fe(III) | 5.62 | | 0.106 |
| Aquifer sediment | LDS20 | | Burk | 51.58 | | 0.038 |
|  | LDS20 | | Burk | 53.84 | | 0.039 |
|  | LDS20 | | Burk | 54.56 | | 0.040 |
|  | LDS20 | | Burk+Fe(III) | 46.68 | | 0.063 |
|  | LDS20 | | Burk+Fe(III) | 43.42 | | 0.080 |
|  | LDS20 | | Burk+Fe(III) | 46.49 | | 0.076 |
|  | XHC | | Burk | 9.64 | | 0.043 |
|  | XHC | | Burk | 9.61 | | 0.043 |
|  | XHC | | Burk | 9.94 | | 0.044 |
|  | XHC | | Burk+Fe(III) | 12.56 | | 0.045 |
|  | XHC | | Burk+Fe(III) | 12.28 | | 0.045 |
|  | XHC | | Burk+Fe(III) | 13.24 | | 0.048 |
|  | LDS30 | | Burk | 32.24 | | 0.036 |
|  | LDS30 | | Burk | 33.01 | | 0.038 |
|  | LDS30 | | Burk | 32.64 | | 0.037 |
|  | LDS30 | | Burk+Fe(III) | 40.85 | | 0.041 |
|  | LDS30 | | Burk+Fe(III) | 40.21 | | 0.040 |
|  | LDS30 | | Burk+Fe(III) | 38.61 | | 0.045 |
| Hot spring sediment | DB-A-1 | | Burk | 0.006 | | 0.599 |
|  | DB-A-1 | | Burk | 0.005 | | 0.514 |
|  | DB-A-1 | | Burk | 0.006 | | 0.509 |
|  | DB-A-1 | | Burk+Fe(III) | 0.003 | | 0.864 |
|  | DB-A-1 | | Burk+Fe(III) | 0.004 | | 0.980 |
|  | DB-A-1 | | Burk+Fe(III) | 0.003 | | 0.850 |
|  | DB-A-3 | | Burk | 0.130 | | 0.168 |
|  | DB-A-3 | | Burk | 0.127 | | 0.167 |
|  | DB-A-3 | | Burk | 0.131 | | 0.169 |
|  | DB-A-3 | | Burk+Fe(III) | 0.142 | | 0.164 |
|  | DB-A-3 | | Burk+Fe(III) | 0.136 | | 0.170 |
|  | DB-A-3 | | Burk+Fe(III) | 0.149 | | 0.170 |
|  | DB-B-3 | | Burk | 0.015 | | 0.111 |
|  | DB-B-3 | | Burk | 0.016 | | 0.112 |
|  | DB-B-3 | | Burk | 0.015 | | 0.150 |
|  | DB-B-3 | | Burk+Fe(III) | 0.018 | | 0.104 |
|  | DB-B-3 | | Burk+Fe(III) | 0.017 | | 0.133 |
|  | DB-B-3 | | Burk+Fe(III) | 0.019 | | 0.141 |

**Table S7** Information on medium-quality metagenome-assembled genomes (MAGs) containing nitrogen fixation and ferric iron reduction genes in environmental samples.

| **Sample** | **Bin name** | **Completeness (%)** | **Contamination (%)** | | **Classification** | **Abundance**  **(Log_10_(TPM+1))** | |
| --- | --- | --- | --- | --- | --- | --- | --- |
| Groundwater | GW.bins.16 | 68.59 | 1.41 | *d__Bacteria;p__Nitrospirota;c__Thermodesulfovibrionia;o__Thermodesulfovibrionales* | | | 2.78 |
| Sediment | S.bins.31 | 97.83 | 0.43 | *d__Archaea;p__Thermoplasmatota;c__Thermoplasmata* | | | 1.45 |
|  | S.bins.126 | 64.05 | 13.15 | *d__Bacteria;p__Pseudomonadota;c__Gammaproteobacteria;o__Burkholderiales;f__Rhodocyclaceae* | | | 1.48 |
| Hot spring | Daba-bins.5 | 72.8 | 8.55 | *d__Bacteria;p__Chloroflexota;c__Anaerolineae;o__Promineifilales;f__Promineifilaceae* | | | 1.50 |
|  | Daba-bins.8 | 72.81 | 4.46 | *d__Bacteria;p__Bacteroidota;c__Bacteroidia;o__Chitinophagales;f__Saprospiraceae* | | | 1.38 |
|  | Daba-bins.12 | 98.74 | 0.94 | *d__Bacteria;p__Bacteroidota;c__Bacteroidia;o__Chitinophagales;f__Saprospiraceae* | | | 0.71 |
|  | Daba-bins.65 | 99.69 | 0.92 | *d__Bacteria;p__Chloroflexota;c__Chloroflexia;o__Chloroflexales;f__Roseiflexaceae;g__Roseiflexus* | | | 0.70 |
|  | Daba-bins.93 | 72.2 | 3.42 | *d__Bacteria;p__Actinomycetota;c__Actinomycetes* | | | 0.84 |
|  | Daba-bins.96 | 76.75 | 0.92 | *d__Bacteria;p__Pseudomonadota;c__Gammaproteobacteria;o__Enterobacterales;f__Aeromonadaceae;g__Aeromonas* | | | 1.03 |
|  | Daba-bins.118 | 67.5 | 3.24 | *d__Bacteria;p__Actinomycetota;c__Actinomycetes* | | | 1.32 |
|  | Daba-bins.146 | 98.18 | 6.36 | *d__Bacteria;p__Chloroflexota;c__Anaerolineae;o__Anaerolineales;f__Villigracilaceae* | | | 0.49 |
|  | Dazuo-bins.13 | 73.13 | 1.07 | *d__Bacteria;p__Cyanobacteriota;c__Cyanobacteriia;o__Thermostichales;f__Thermostichaceae;g__Thermostichus* | | | 0.61 |
|  | Dazuo-bins.46 | 98.09 | 1.14 | *d__Bacteria;p__Bacteroidota;c__Bacteroidia* | | | 6.51 |
|  | Dazuo-bins.47 | 59.88 | 8.36 | *d__Bacteria;p__Chloroflexota;c__Anaerolineae;o__Anaerolineales* | | | 0.35 |
|  | Dazuo-bins.59 | 64.08 | 2.27 | *d__Bacteria;p__Chloroflexota;c__Anaerolineae;o__Anaerolineales;f__Anaerolineaceae;g__Bellilinea* | | | 0.52 |
|  | Dazuo-bins.78 | 95.33 | 0 | *d__Bacteria;p__Aquificota;c__Aquificae;o__Hydrogenothermales;f__Hydrogenothermaceae;g__Sulfurihydrogenibium* | | | 1.17 |
| Soil | soil2.bins.12 | 98.28 | 2.89 | Unclassified Archaea | | | 0.78 |
|  | Soil4.bins.12 | 70.18 | 1.92 | *d__Archaea;p__Thermoplasmatota;c__SW-10-69-26* | | | 0.88 |
| Marine | sea2.bins.6 | 96.94 | 2.17 | *d__Bacteria;p__Pseudomonadota;c__Alphaproteobacteria;o__Sphingomonadales;f__Sphingomonadaceae* | | | 1.38 |
|  | sea4.bins.36 | 55.52 | 1.72 | Unclassified Bacteria | | | 1.13 |
|  | sea8.bins.14 | 98.32 | 4.15 | Unclassified Bacteria | | | 1.08 |
|  | sea8.bins.19 | 77.05 | 1.01 | *d__Bacteria;p__Pseudomonadota;c__Gammaproteobacteria;o__Enterobacterales;f__Neiellaceae* | | | 0.38 |
|  | sea8.bins.11 | 59.09 | 2.93 | *d__Bacteria;p__Bacteroidota;c__Bacteroidia;o__Bacteroidales;f__Prolixibacteraceae* | | | 0.44 |
|  | sea8.bins.3 | 58.01 | 3.51 | *d__Bacteria;p__Desulfobacterota;c__Desulfuromonadia;o__Desulfuromonadales* | | | 1.32 |
|  | sea8.bins.5 | 54.20 | 3.57 | *d__Bacteria;p__Desulfobacterota;c__Desulfobulbia;o__Desulfobulbales;f__Desulfocapsaceae* | | | 4.03 |

**Table S8** Estimation of nitrogen fixation enhancement through ferric iron reduction in environmental samples.

|  | Samples | Fe(Ⅲ) addition in microcosm  (mmol L^-1^) | Promoted ^15^N fixation  (μmol L^-1^) | Contribution  (μmol N/mmol Fe) |
| --- | --- | --- | --- | --- |
| Aquifer  sediments | X2 | 0.2 | 11.39 | 56.94 |
|  | L2 | 0.2 | 89.67 | 448.37 |
|  | L3 | 0.2 | 33.49 | 167.45 |
| Soils | JH1 | 0.2 | 12.11 | 60.56 |
|  | TSH | 0.2 | 5.31 | 26.55 |
|  | S07 | 0.2 | 5.92 | 29.59 |
| Marine sediments | A7 | 0.2 | 4.41 | 22.04 |
|  | A7-7 | 0.2 | 16.73 | 83.64 |
|  | C4 | 0.2 | 2.31 | 11.54 |

- Values presented in the table represent mean values.
- The data is calculated from the results of ^15^N isotope tracing of environmental samples.


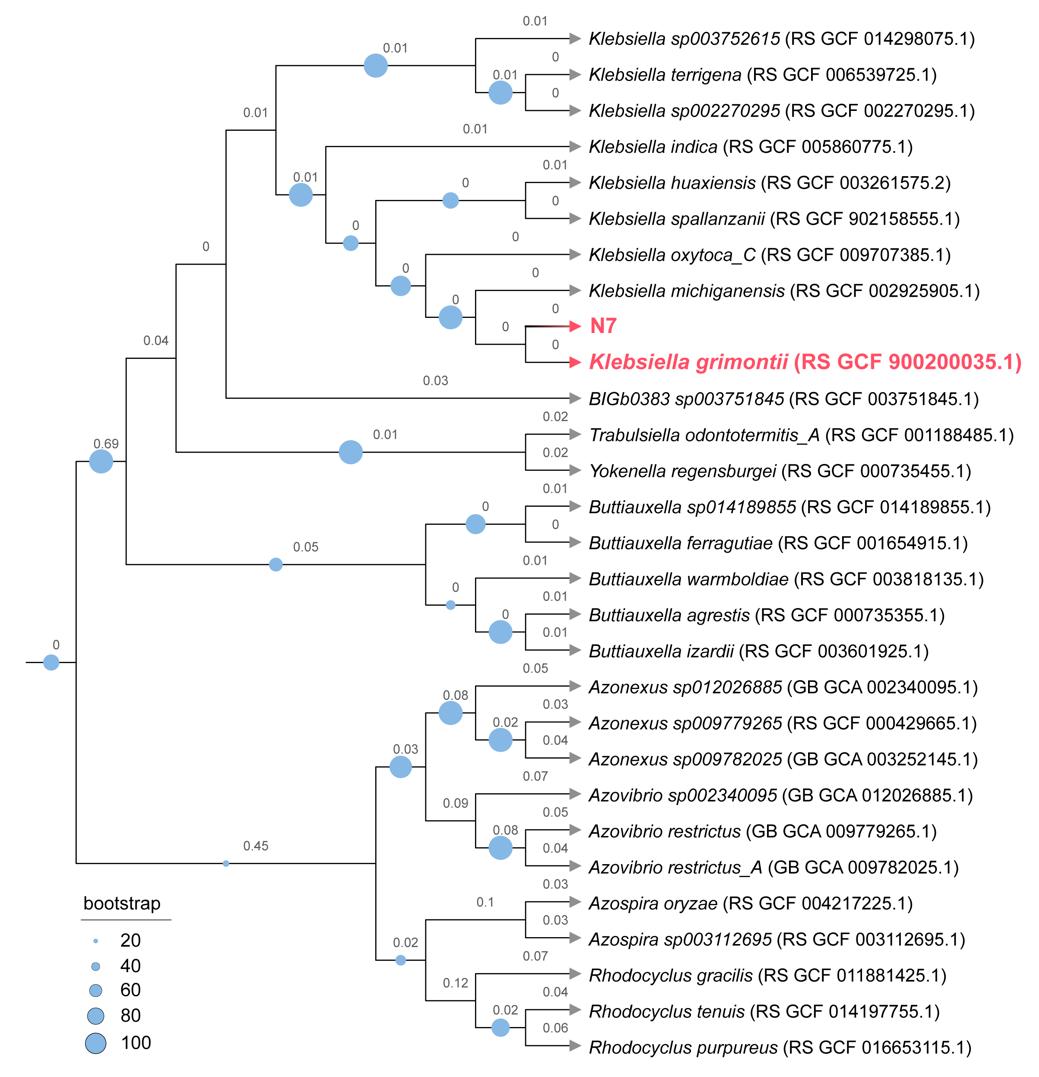


**Fig. S1 Phylogenetic analysis of diazotroph N7 based on GTDB-Tk (maximum likelihood method).** A phylogenetic tree of the target genome and reference genomes was constructed using 120 core single-copy genes, and 28 genomes closely related to the N7 genome were selected. Branch support values (Bootstrap) are labeled at the tree nodes.


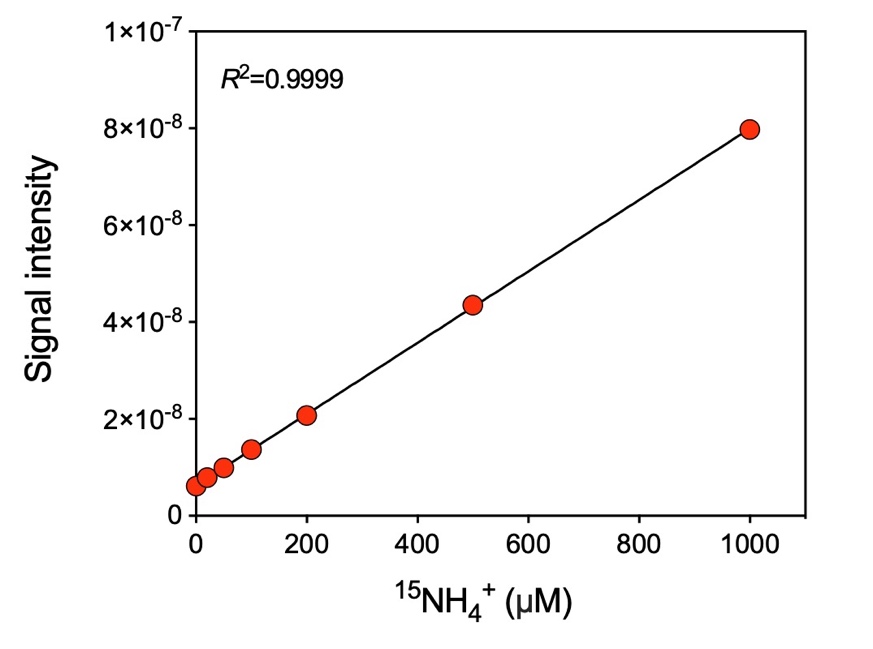


**Fig. S2 Relationships between known ^15^NH_4_^+^ concentrations and measured signal intensities of total** **^15^N_2_ (^29^N_2_ +2× ^30^N_2_).** The concentration of the generated ^15^N_2_ is measured with membrane inlet mass spectrometry (MIMS). The values in the figure are the average of three parallel samples.

**
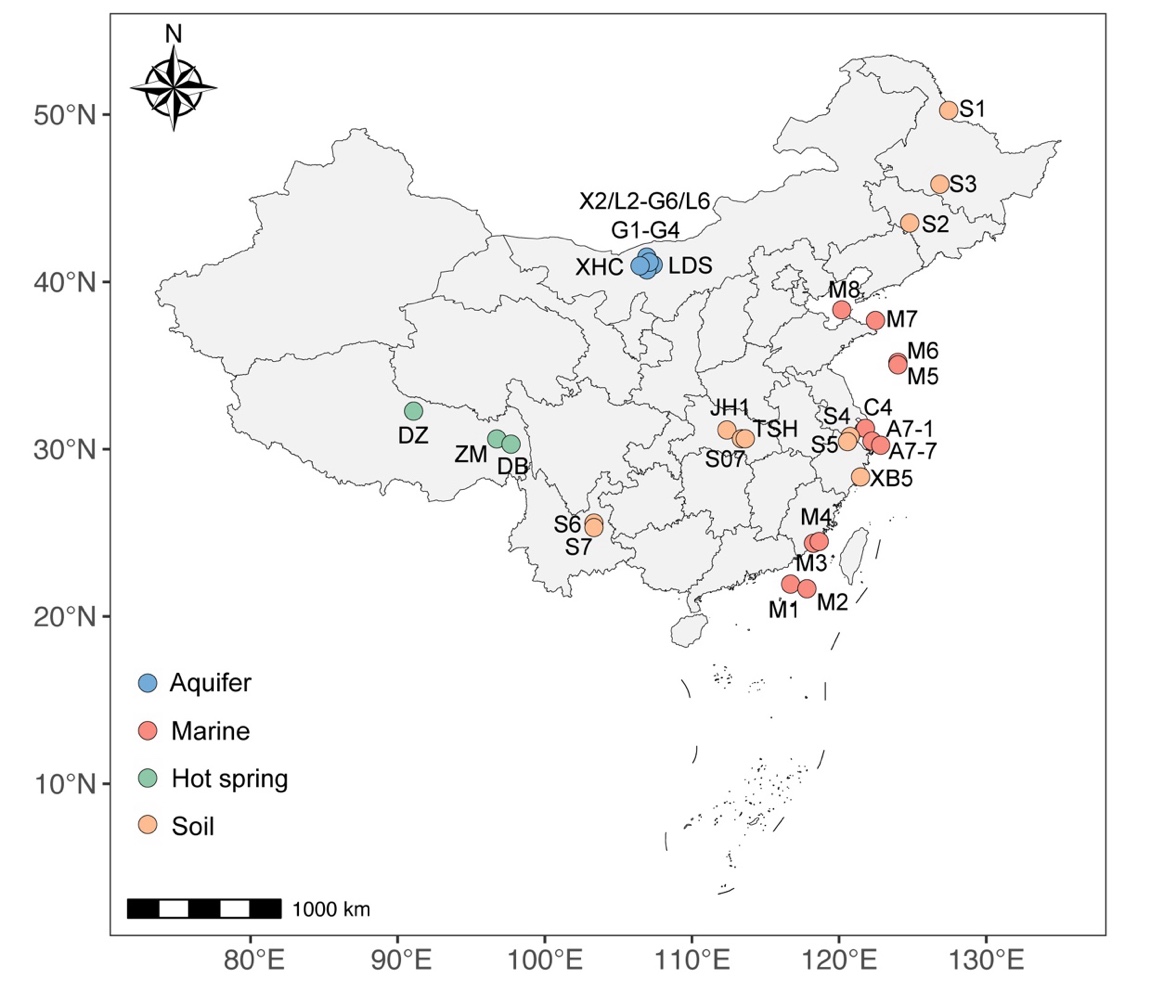
**

**Fig. S3 Geographic distribution of environmental sampling sites in China, where microcosm, metagenomic, and metatranscriptomic data were collected.** The samples were taken from aquifer waters/sediments (blue dots), marine sediments (red dots), hot spring sediments (green dots), and soils (orange dots).


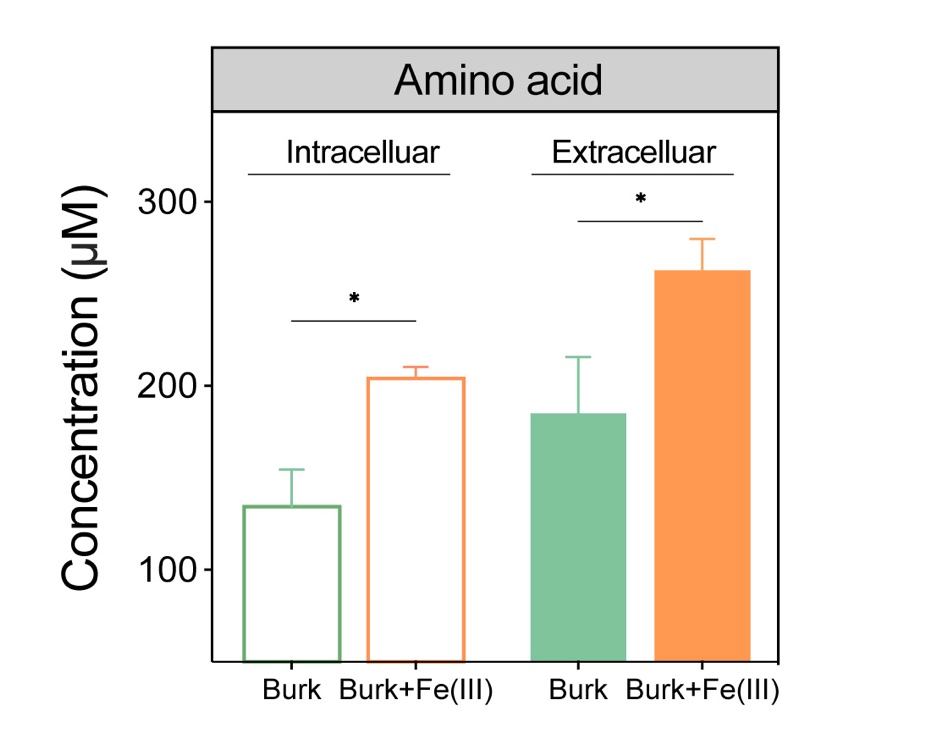


**Fig. S4 Amino acid concentrations in *Klebsiella grimontii* N7 with or without ferric citrate.** * represents *P* < 0.05.


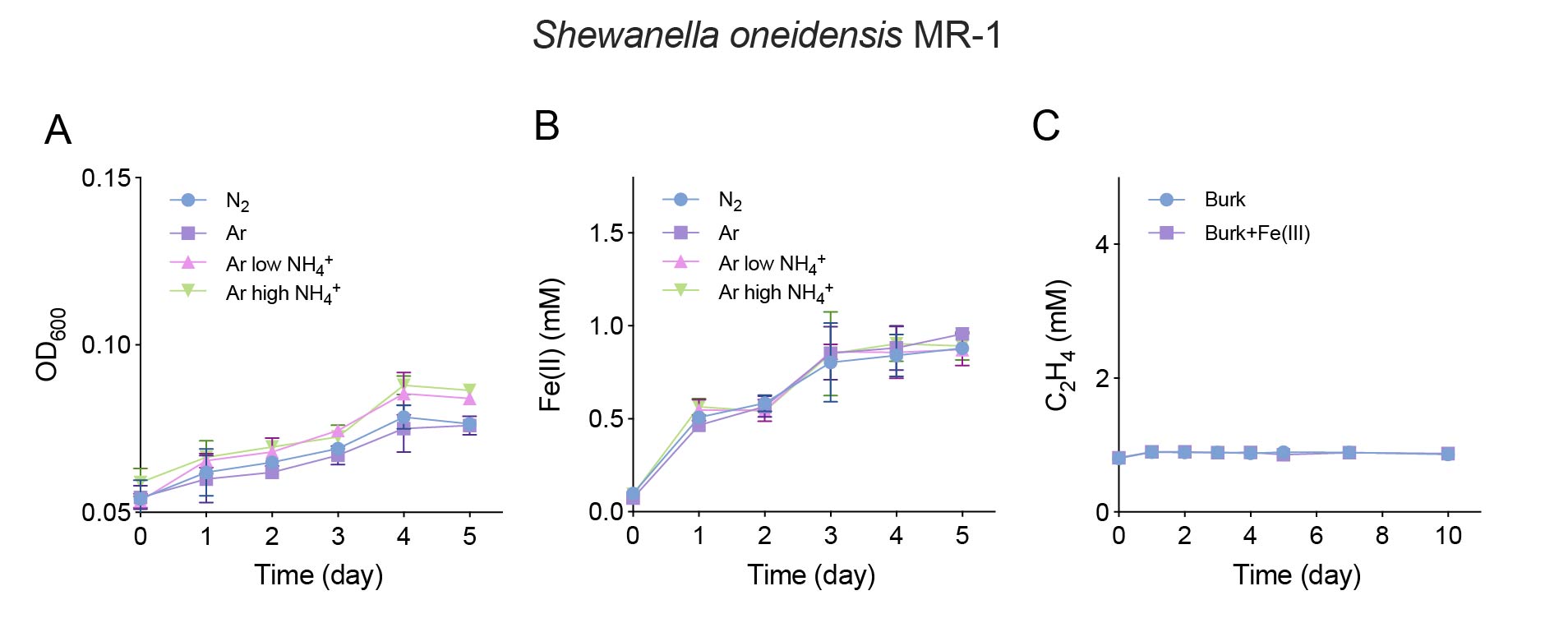


**Fig. S5 Nitrogenase activity of strain *Shewanella oneidensis* MR-1 in nitrogen-free medium.**

**Fig. S6 Nitrogenase activity of strains *Azospirillum humicireducens* SgZ-5T and *Shewanella* *oneidensis* MR-1 co-cultures under nitrogen and Fe(III)-free conditions.**


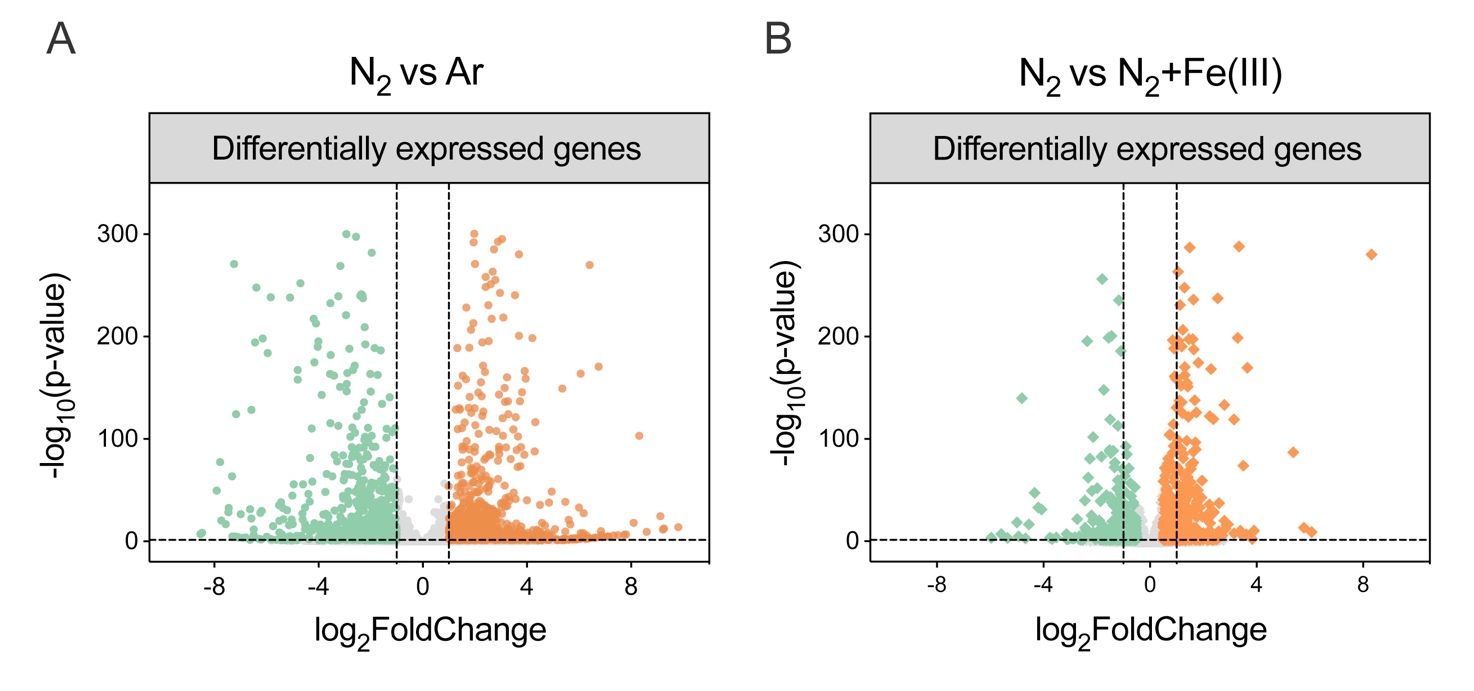


**Fig. S7 Volcano map of differential expression genes of strain *Klebsiella grimontii* N7 with or without the addition of (A) N_2_ and (B) Fe(III).**


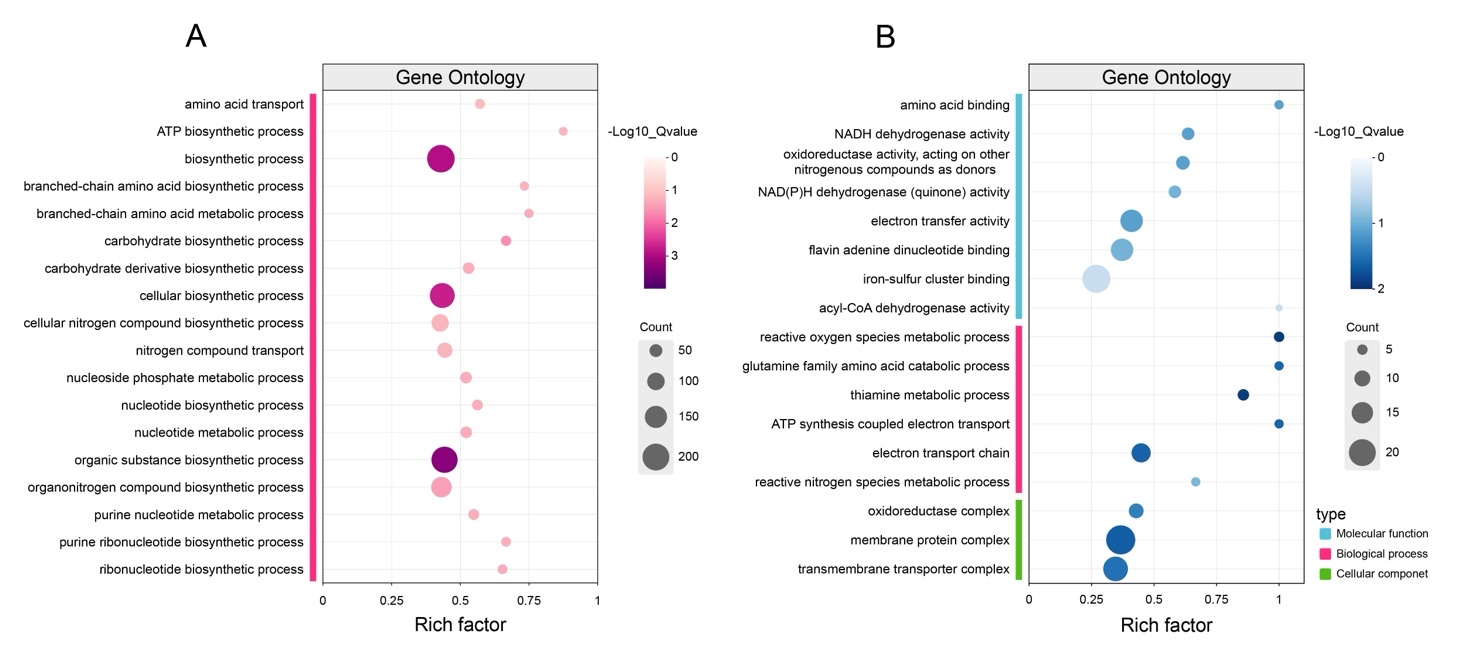


**Fig. S8 Enrichment analysis of GO functional categories in strain** ***Klebsiella* *grimontii* N7 under different conditions.** (A) GO enrichment in strain *Klebsiella* *grimontii* N7 under N_2_ versus Ar conditions. (B) GO enrichment in strain *Klebsiella* *grimontii* N7 with or without ferric iron addition.


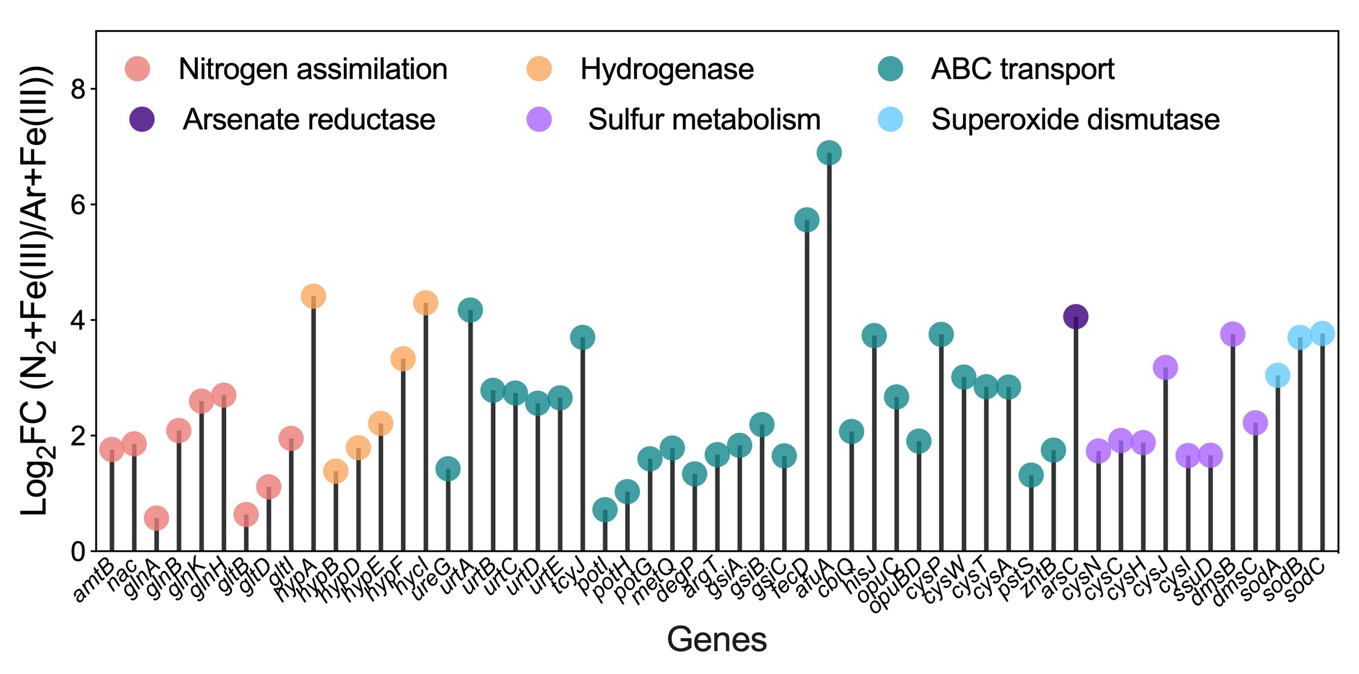


**Fig. S9** **Differentially expressed gene levels in strain** ***Klebsiella* *grimontii* N7** **with or without N_2_ addition (*****P* < 0.01).** Upregulated genes were measured by log_2_-fold change (Log_2_FC) under N_2_+Fe(III) compared to Ar+Fe(III) incubation (*P* < 0.01).

**
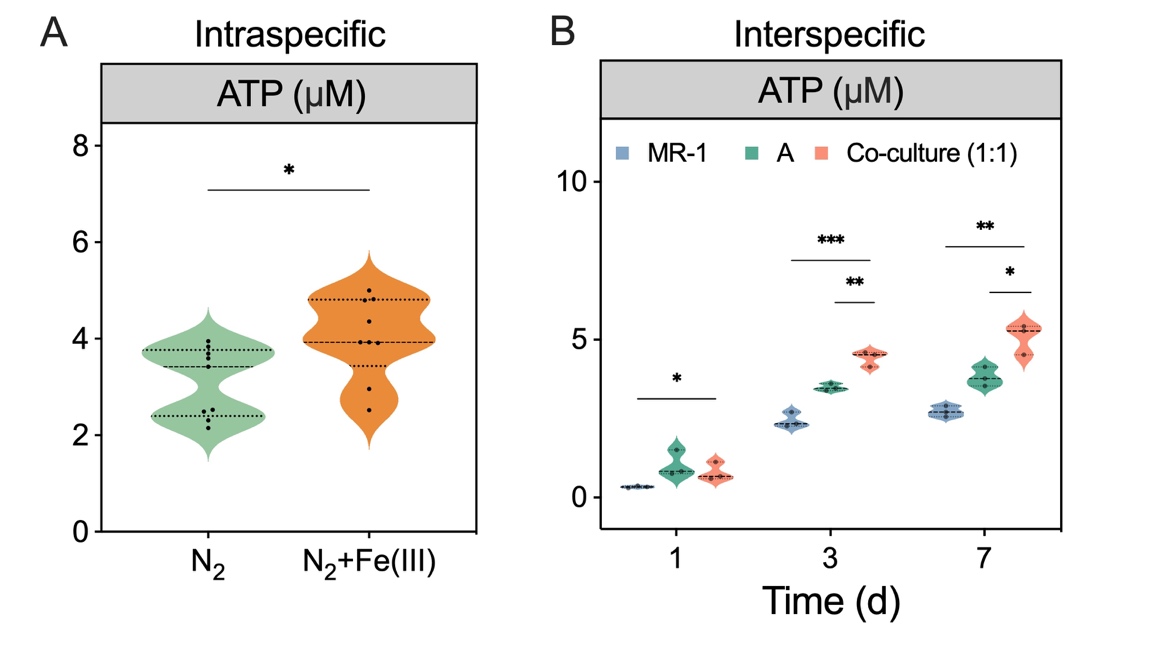
**

**Fig. S10 ATP concentrations in** **(A) *Klebsiella* *grimontii* N7 under ferric iron conditions** **and (B) *Azospirillum humicireducens* SgZ-5T and** ***Shewanella* *oneidensis* MR-1 monocultures and co-culture.** *, **, and *** indicate significant differences at *P* < 0.05, < 0.01, and < 0.001, respectively (t-test).


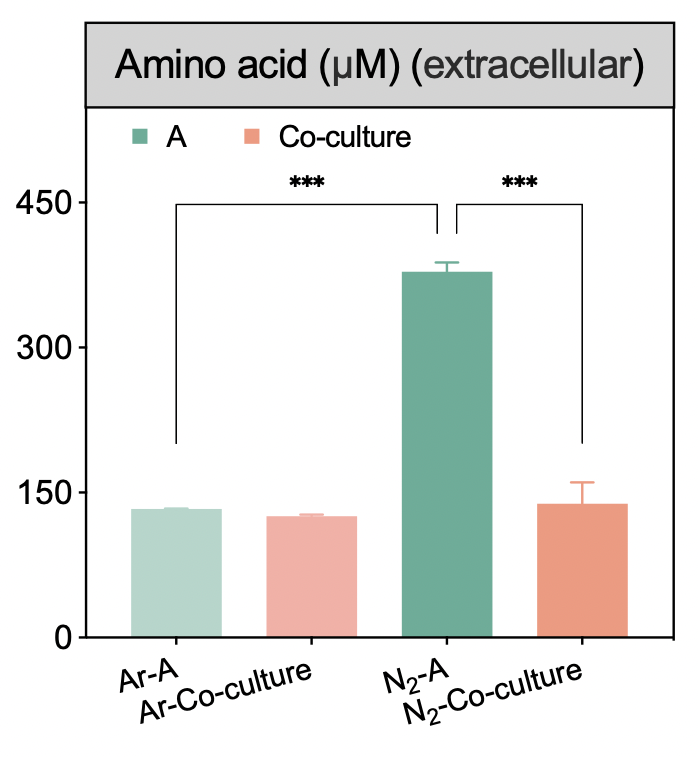


**Fig. S11 Extracellular amino acid concentrations in *Azospirillum humicireducens* SgZ-5T monoculture and co-culture.** *** represent *P* < 0.001.


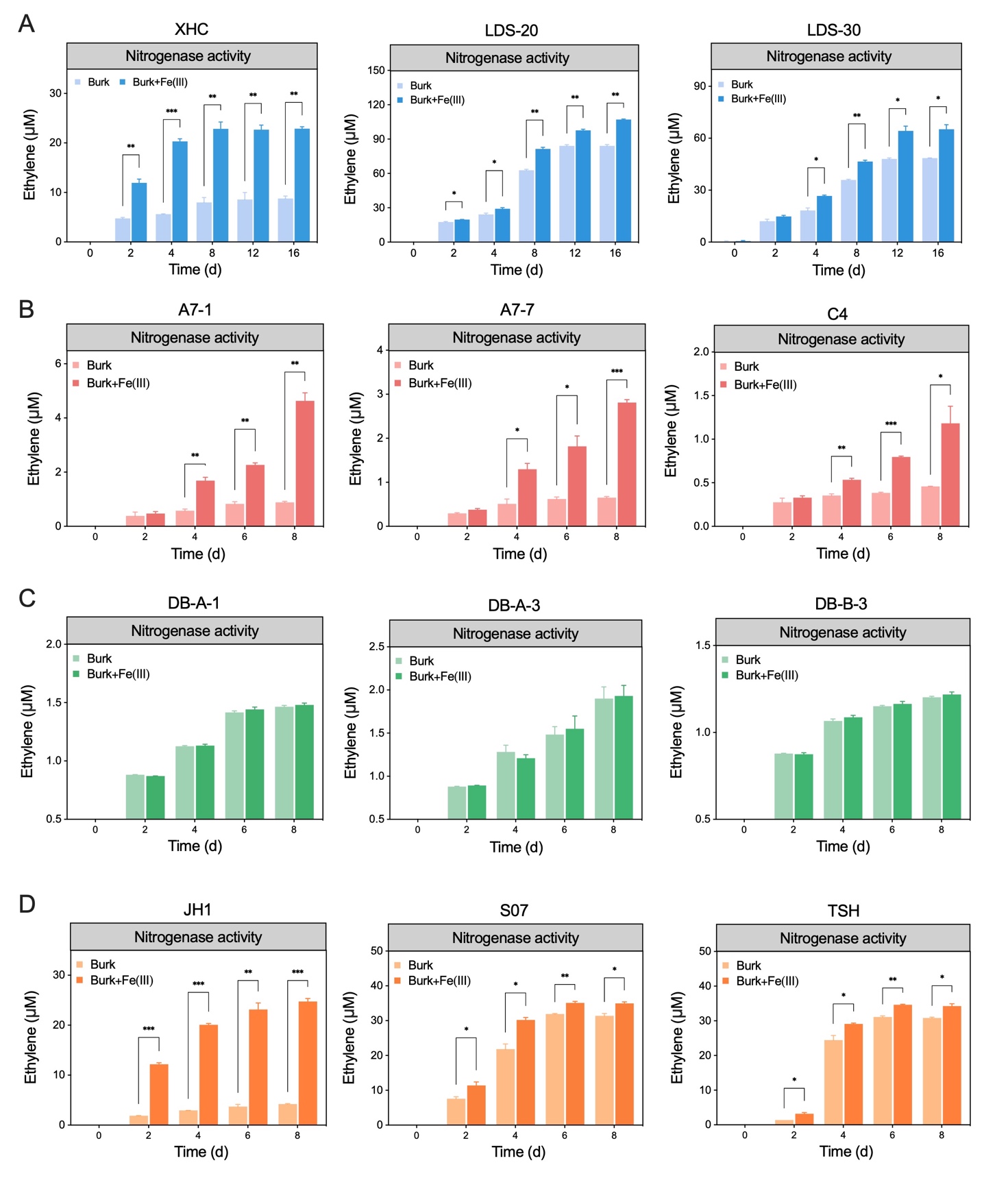


**Fig. S12 Nitrogen fixation activity in (A) aquifer sediments, (B) marine sediments,** **(C) hot spring sediments, and (D) soils**. Burk: no addition of Fe(III); Burk + Fe(III): addition of Fe(III)-citrate. *, **, and *** indicate significant differences at *P* < 0.05, < 0.01, and < 0.001, respectively (t-test).


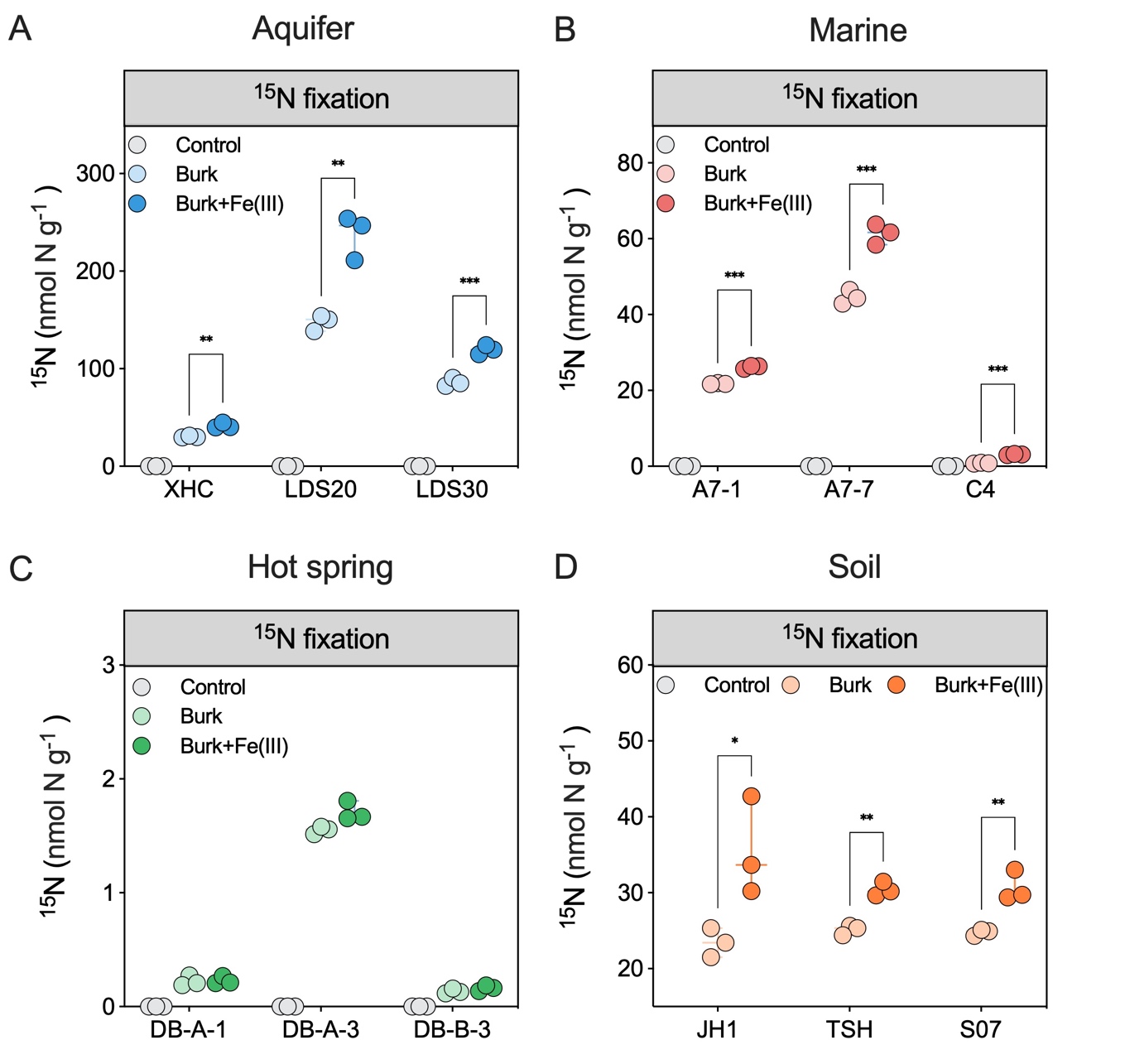


**Fig.** **S13 ^15^N fixation amounts in (A) aquifer sediments, (B) marine sediments,** **(C) hot spring sediments, and (D) soil samples after incubation under conditions with or without Fe(III) addition.** The data were calculated based on δ^15^N (‰) and total nitrogen (%) values determined by isotope ratio mass spectrometry. *, **, and *** indicate significant differences at *P* < 0.05, < 0.01, and < 0.001, respectively (t-test).


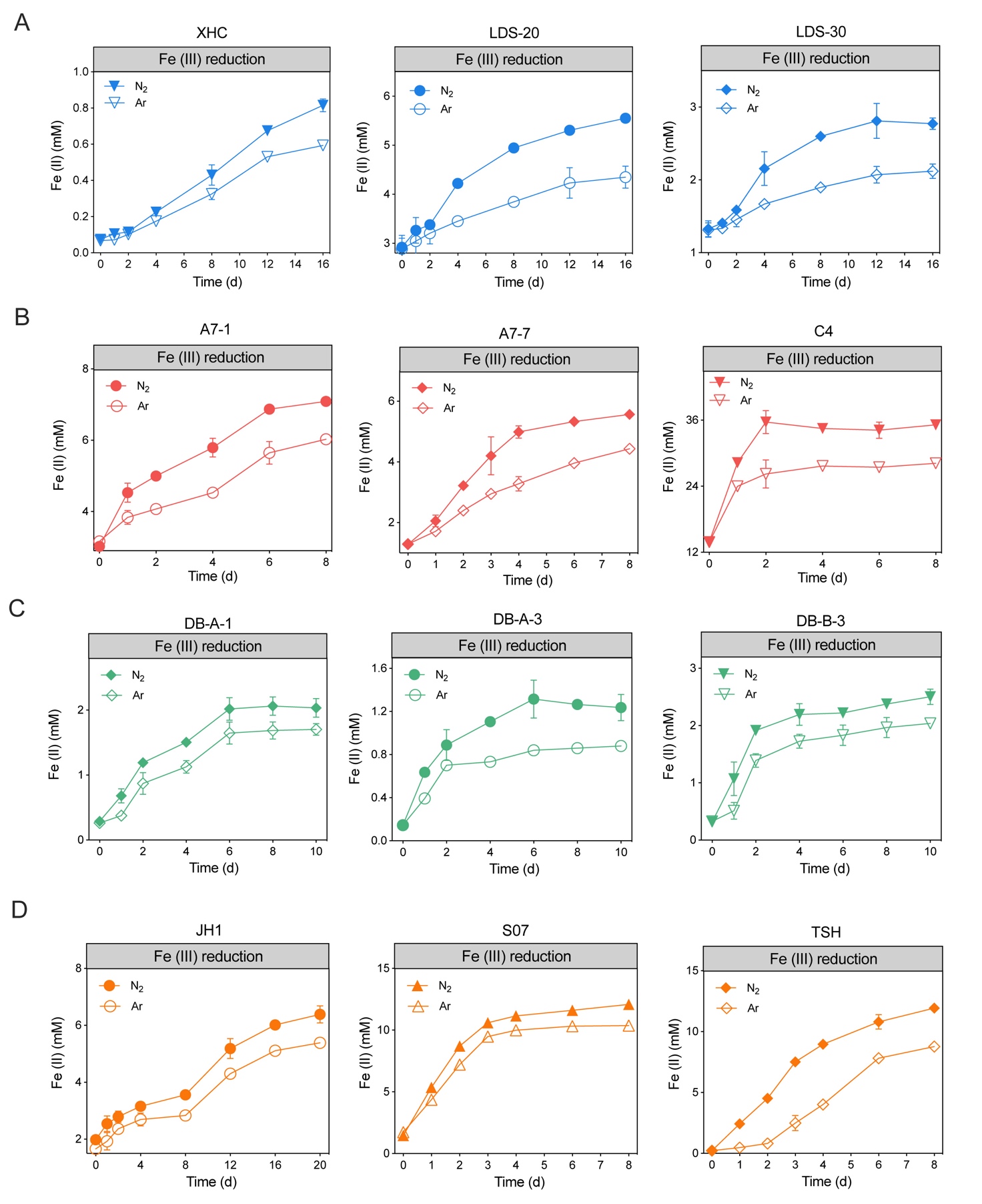


**Fig. S14 Iron reduction kinetics in (A) aquifer sediments, (B) marine sediments, (C) hot spring sediments, and (D) soils under N_2_ and Ar conditions.**


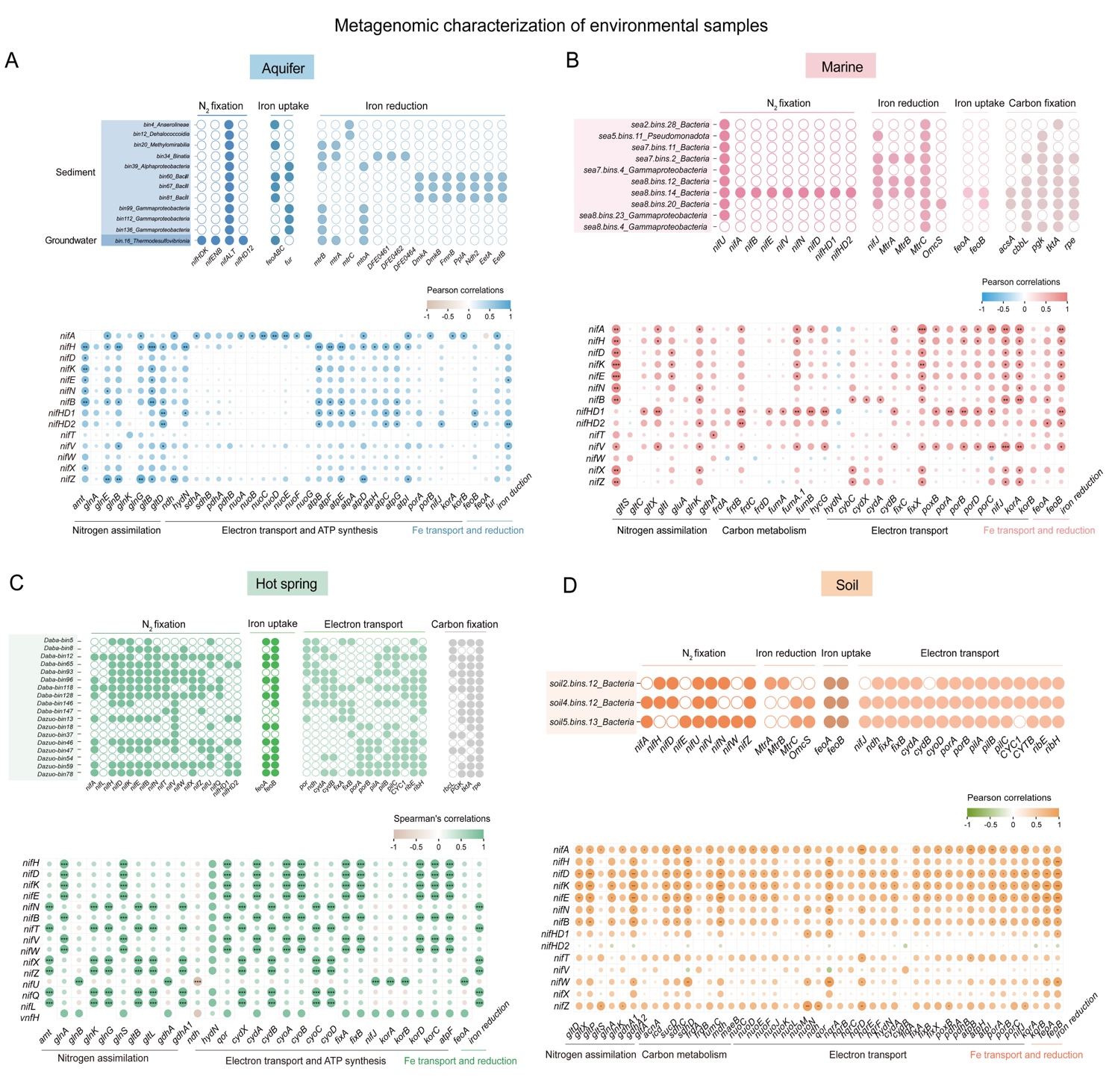


**Fig.** **S15 Metagenomic analysis of environmental samples from (A) aquifer waters/sediments, (B) marine waters/sediments, (C) hot spring sediments, and (D) soils.** The figure includes the correlations between nitrogen fixation genes and functional genes involved in nitrogen transport/assimilation, carbon metabolism, and dissimilatory iron reduction. Metagenome-assembled genomes (MAGs) reconstructed from aquifer, marine, hot spring, and soil environments show the co-occurrence of genes related to nitrogen fixation and Fe(III) reduction.


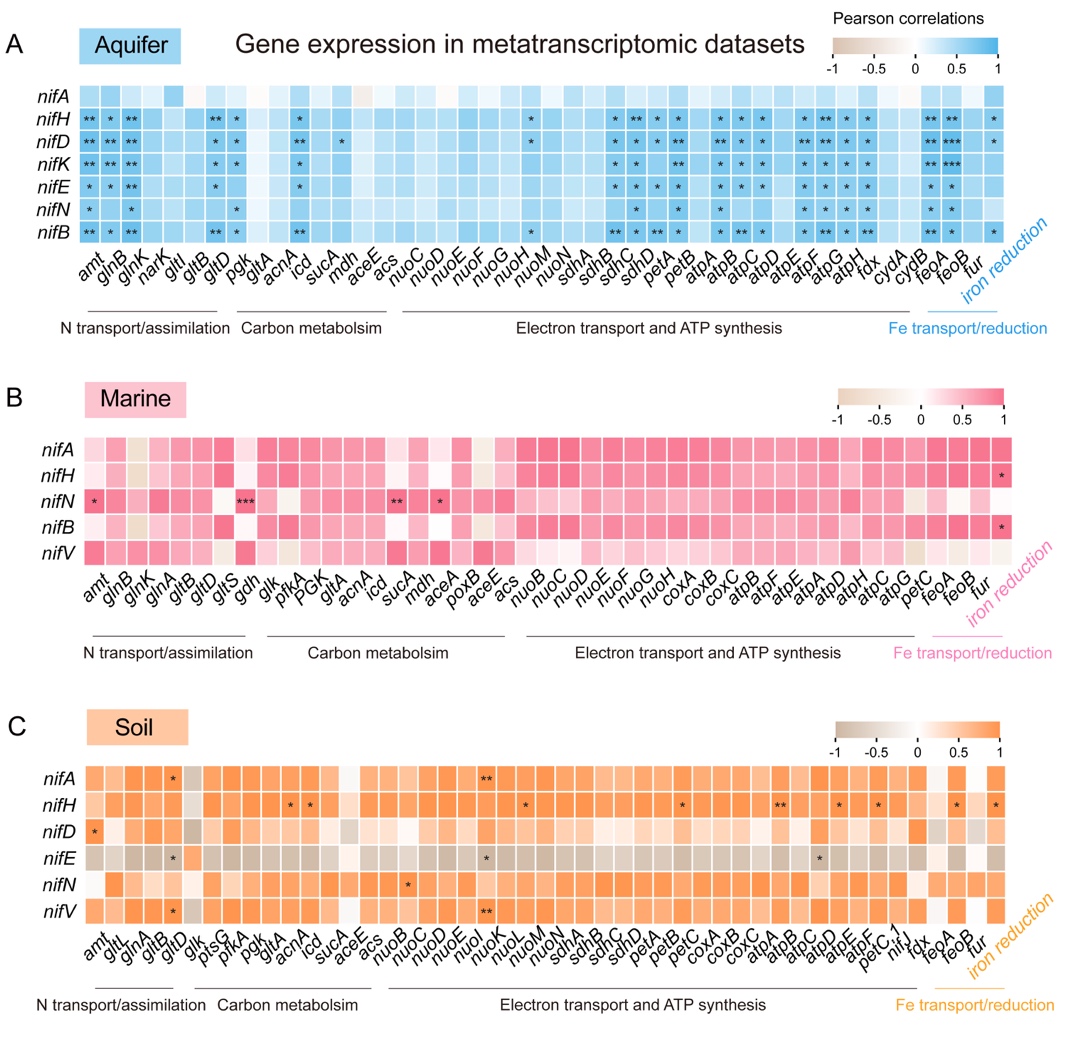


**Fig. S16 Correlations between nitrogen fixation genes and functional genes related to nitrogen transport/assimilation, carbon metabolism, and dissimilatory iron reduction in (A) aquifer waters/sediments, (B) marine sediments, and (C) soils based on metatranscriptomic analysis.**

**
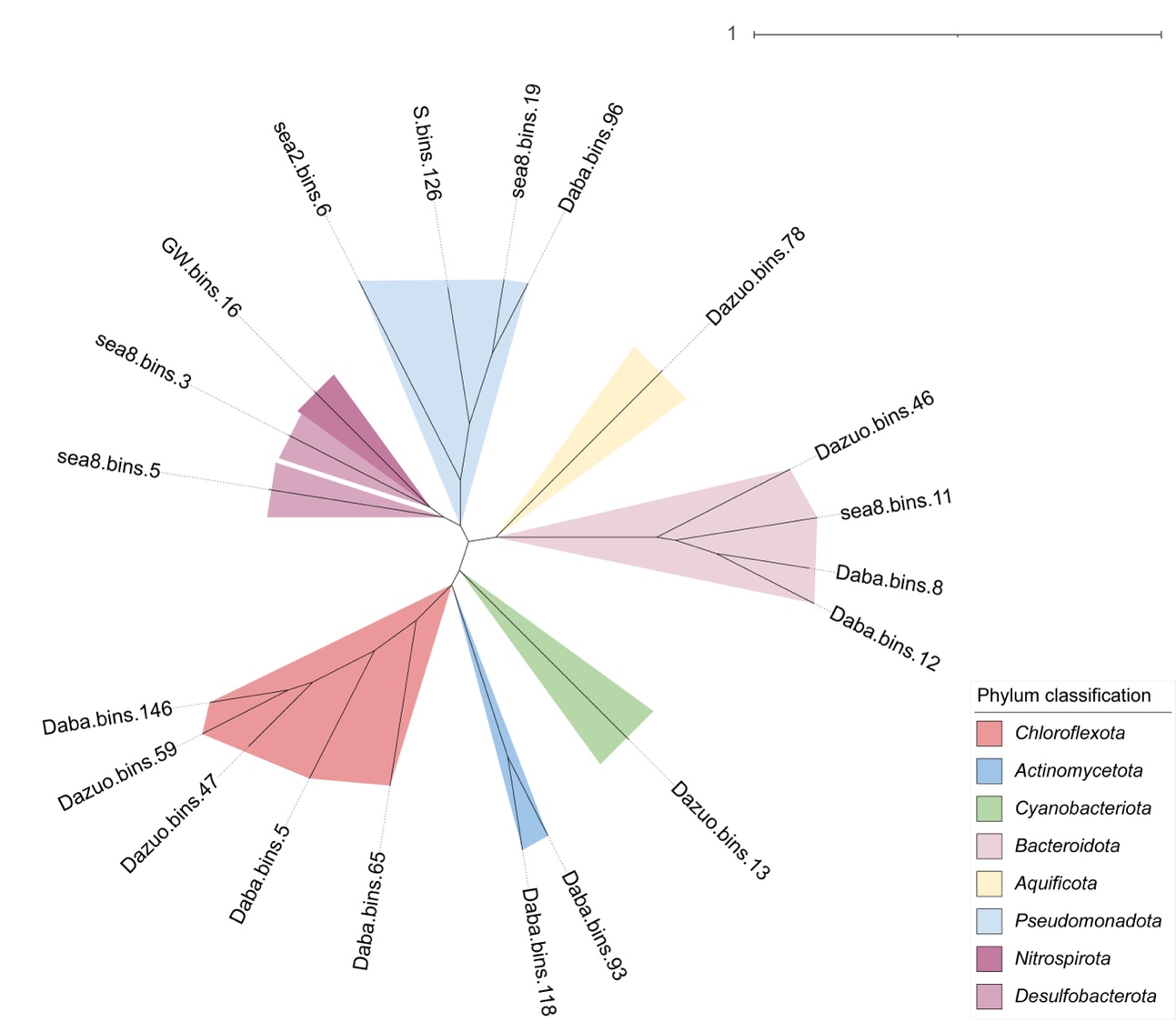
**

**Fig. S17 Phylogenetic tree of 20 bacterial genome bins containing nitrogen fixation and ferric iron reduction genes in environmental samples.** The scale bar represents 1 nucleotide substitutions per site.


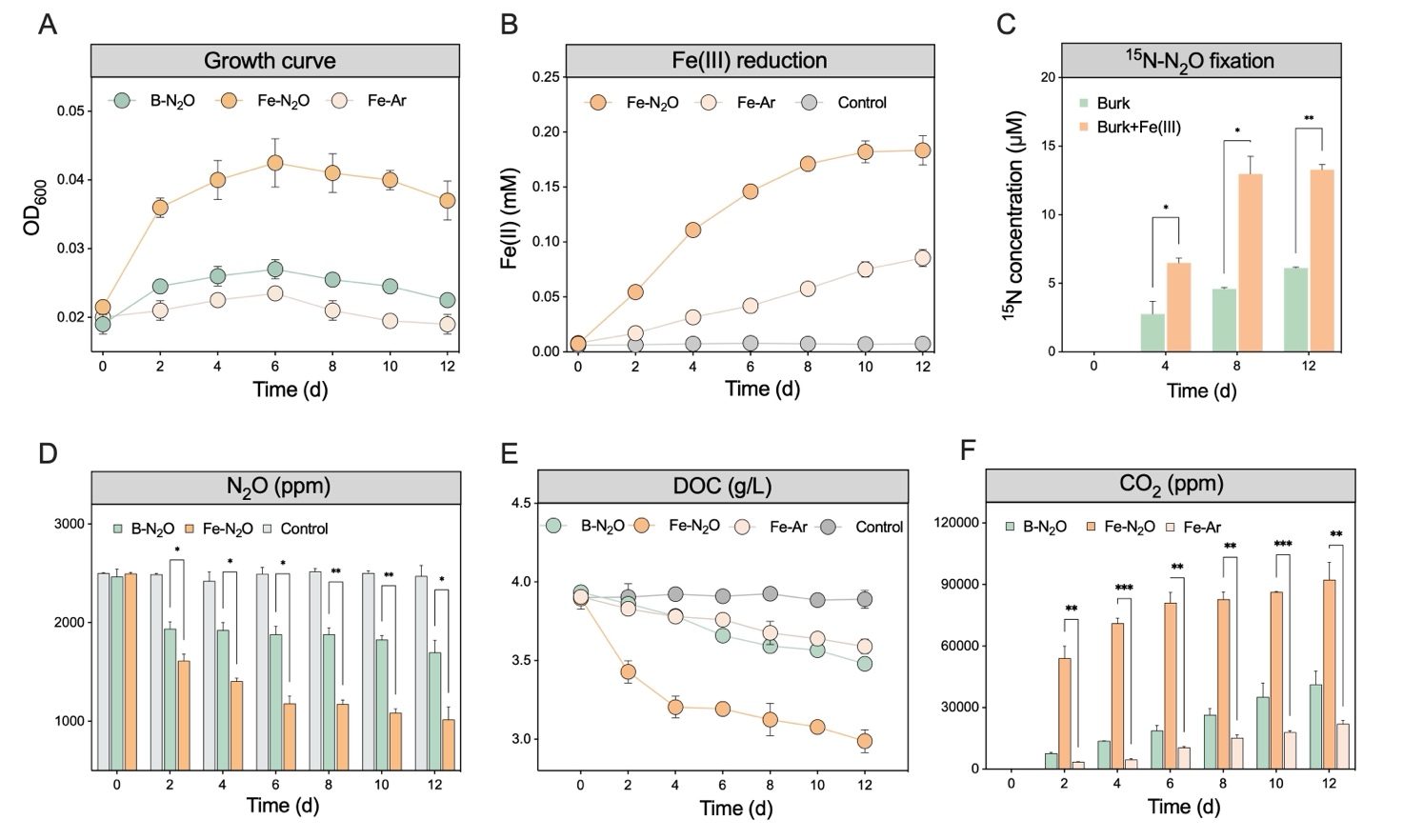


**Fig. S18 The synergistic interaction between N_2_O fixation and Fe(III) reduction in diazotrophic iron-reducing bacterium** ***Klebsiella grimontii* N7.** Effects of N_2_O fixation on (A) growth and (B) Fe(Ⅲ) reduction in N7 cultures. (C) ^15^N-N_2_O fixation, (D) N_2_O consumption, (E) DOC consumption, and (F) CO_2_ production in *K. grimontii* N7 with or without ferric citrate. *, **, and *** represent *P* < 0.05, 0.01, and 0.001, respectively.

**Reference**

1. Sigman D, Casciotti K, Andreani M *et al*. A bacterial method for the nitrogen isotopic analysis of nitrate in seawater and freshwater. Anal. Chem. 2001; 73:4145-53.

2. Bolger A, Lohse M, Usadel B. Trimmomatic: A flexible trimmer for illumina sequence data. Bioinformatics. 2014; 30:2114-20.

3. Nurk S, Meleshko D, Korobeynikov A *et al*. metaSPAdes: a new versatile metagenomic assembler. Genome Res. 2017; 27:824-34.

4. Grabherr MG, Haas BJ, Yassour M *et al*. Full-length transcriptome assembly from RNA-Seq data without a reference genome. Nat. Biotechnol. 2011; 29:644-52.

5. Kopylova E, Noé L, Touzet H. SortMeRNA: Fast and accurate filtering of ribosomal RNAs in metatranscriptomic data. Bioinformatics 2012; 28:3211-17.

6. Hyatt D, LoCascio PF, Hauser LJ *et al*. Gene and translation initiation site prediction in metagenomic sequences. Bioinformatics. 2012; 28:2223-30.

7. Patro R, Duggal G, Love MI *et al*. Salmon provides fast and bias-aware quantification of transcript expression. Nat. Methods. 2017; 14:417-19.

8. Aramaki T, Blanc-Mathieu R, Endo H *et al*. KofamKOALA: KEGG Ortholog assignment based on profile HMM and adaptive score threshold. Bioinformatics. 2020; 36:2251-52.

9. Garber AI, Nealson KH, Okamoto A *et al*. FeGenie: A comprehensive tool for the identification of iron genes and iron gene neighborhoods in genome and metagenome assemblies. Front. Microbiol. 2020; 11::37.

10. Uritskiy GV, DiRuggiero J, Taylor J. MetaWRAP—a flexible pipeline for genome-resolved metagenomic data analysis. Microbiome. 2018; 6:158.

11. Liu L, Wang Y, Yang Y *et al*. Charting the complexity of the activated sludge microbiome through a hybrid sequencing strategy. Microbiome. 2021; 9:205.

12. Parks DH, Imelfort M, Skennerton CT *et al*. CheckM: assessing the quality of microbial genomes recovered from isolates, single cells, and metagenomes. Genome Res. 2014; 25:1043-55.

13. Chaumeil P-A, Mussig AJ, Hugenholtz P *et al*. GTDB-Tk: a toolkit to classify genomes with the Genome Taxonomy Database. Bioinformatics. 2020; 36:1925-27.
